# An Optimized Probabilistic Roadmap Algorithm for Path Planning of Mobile Robots in Complex Environments with Narrow Channels

**DOI:** 10.3390/s22228983

**Published:** 2022-11-20

**Authors:** Lijun Qiao, Xiao Luo, Qingsheng Luo

**Affiliations:** 1 School of Mechatronical Engineering, Beijing Institute of Technology, Beijing 100081, China; 2 School of Computer Science and Technology, Beijing Institute of Technology, Beijing 100081, China

**Keywords:** path planning, narrow channels, probabilistic road-map algorithm, path pruning technology, artificial potential function

## Abstract

In this paper, we propose a new path planning algorithm based on the probabilistic roadmaps method (PRM), in order to effectively solve the autonomous path planning of mobile robots in complex environments with multiple narrow channels. The improved PRM algorithm mainly improves the density and distribution of sampling points in the narrow channel, through a combination of the learning process of the PRM algorithm and the APF algorithm. We also shortened the required time and path length by optimizing the query process. The first key technology to improve the PRM algorithm involves optimizing the number and distribution of free points and collision-free lines in the free workspace. To ensure full visibility of the narrow channel, we extend the obstacles through the diagonal distance of the mobile robot while ignoring the safety distance. Considering the safety distance during movement, we re-classify the all sampling points obtained by the quasi-random sampling principle into three categories: free points, obstacle points, and adjacent points. Next, we transform obstacle points into the free points of the narrow channel by combining the APF algorithm and the characteristics of the narrow channel, increasing the density of sampling points in the narrow space. Then, we include potential energy judgment into the construction process of collision-free lines shortening the required time and reduce collisions with obstacles. Optimizing the query process of the PRM algorithm is the second key technology. To reduce the required time in the query process, we adapt the bidirectional A* algorithm to query these local paths and obtain an effective path to the target point. We also combine the path pruning technology with the potential energy function to obtain a short path without collisions. Finally, the experimental results demonstrate that the new PRM path planning technology can improve the density of free points in narrow spaces and achieve an optimized, collision-free path in complex environments with multiple narrow channels.

## 1. Introduction

Automatic path planning is an indispensable technology for realizing the intelligence of mobile robots [1,2], which is mainly used to automatically plan a collision-free path for the mobile robot from its initial position to a final position in a complex environment. With the improvement of artificial intelligence in mobile robots, the specific requirements for the final path are gradually increasing, including smooth performance [3], shortest distance parameters [4], and minimum energy parameters [5]. However, finding an effective path without collision in the presence of obstacles has become the foundation of the later research, especially regarding complex workspaces with multiple narrow channels [6,7,8]. Therefore, the path planning through narrow channels has become a key focus of various researchers.

Many researchers have proposed various algorithms to solve path planning problems in complex environments. In the early days, the workspace was divided into grids to find an available path. A* and D* are typical algorithms, but the division into a grid can affect the appearance of obstacles and the direction of movement of the robot [9,10,11]. Meanwhile, inspired by nature, the Artificial Potential Function (APF) algorithm can effectively implement path planning for mobile platforms by constructing the distribution of potential energy fields, including the repulsive and attractive forces in a specified workspace [12,13]. With the development of swarm intelligence technology, researchers have continued to apply different swarm intelligence methods to solve path planning problems, including artificial bee colony [14], genetic algorithms [15], particle swarm optimization [16], and ant colony algorithms [17]. However, compared to other algorithms, these swarm intelligence algorithms may take longer to obtain efficient paths in complex environments. In addition, reinforcement learning methods have been gradually applied to the mobile robot path planning problem [18,19]. However, these algorithms need to obtain position information in real-time and adjust the corresponding state to achieve path planning. Therefore, the algorithm is affected by the performance of its own sensors and the distribution of obstacles. Recently, path planning methods based on random sampling have been widely used and studied, due to their high sampling efficiency and success rate. The most typical methods are rapidly-exploring random trees (RRT) and the probabilistic roadmaps method (PRM), which require use of the sampling points to describe the free space [20,21]. The RRT algorithm randomly selects a free point at each step and uses a separate query process to solve the path planning problem [22]. In contrast, the PRM algorithm constructs the free space and uses a multi-part query method to obtain the path [23]. Compared to the RRT algorithm, the PRM algorithm can quickly achieve a path with the shortest distance by establishing the free space in advance. Therefore, we take the PRM algorithm as the core algorithm and optimize the critical steps of the PRM algorithm by combining the APF algorithm.

The critical steps of the PRM algorithm are divided into two modules: the learning process and the query process [24,25]. The learning process is mainly to construct the free space and obstacle space using different point sets, which are determined by randomly sampling points in the workspace and using collision detection technology [26]. The collision-free line represents the local path, which is composed of the free point set. Therefore, the free space of the specified workspace consists of free points and collision-free lines. The critical step in the path query process is using the local planner to plan the global path without collision in the presence of obstacles. The PRM algorithm can construct the free points and collision-free lines in advance for a specified environment, significantly reducing the required time for collision detection and optimizing the efficiency of path planning [27]. However, after years of in-depth research, the PRM algorithm still has a low success rate for solving path planning problems in complex environments with narrow channels, which is due to the inability of various sampling methods to describe narrow access.

To improve the performance of the PRM algorithm, researchers have proposed various methods to optimize the map construction technology for free space in a complex environment with narrow channels. The first optimization technique proposed the use of various random sampling principles to avoid the inability to achieve full-space distribution, including uniform random sampling technique [28], quasi-random sampling technique [29], and random walk sampling techniques [30]. These random sampling principles mainly increase the density of the sampling points in the narrow channel, according to the distribution of random points. However, it cannot be significantly increased, due to the low proportion of the narrow channel in the overall space. The second approach involves improving the distribution and sampling density in the narrow space, which is the main content of most articles related to optimizing the traditional PRM algorithm. For example, in [31], the Gaussian sampling technique has been proposed to transform the obstacle points into the free points of the obstacle boundary by adding random perturbations. The author of these papers [32,33] has also proposed the bridge sampling technology, which determines the collision-free midpoint position as the sampling point of the narrow passage by sampling between different obstacle points. In [34], Ye has determined the sampling points of narrow passage by using an artificial potential function between different obstacles. The second type of improvement method directly increases the density of free points by sampling near the narrow channel. However, the free points in the narrow channels are limited, due to the large number of obstacles and the significant increase in the surrounding environment. Meanwhile, the free points close to the obstacles increase the probability of collision with the mobile robot.

In order to solve the problem of path planning in complex environments with narrow channels, these above-mentioned studies have mainly focused on how to quickly increase the density of free points in the narrow channel and reduce the collision probability. In this paper, we proposes a new algorithm by effectively combing the APF algorithm and the typical PRM algorithm, in response to the discussed research. The primary process of the new algorithm is based on the PRM algorithm, while the APF algorithm is added to improve the critical operation. Therefore, the new PRM algorithm is not a simple combination of the two different path planning algorithms. The specific contributions of this paper are as follows:

(1) To ensure the existence of narrow passages and optimize the number and distribution of free points in the free workspace, we extend the obstacle based on only the diagonal distance of the mobile robot, ignoring the safety distance in the movement. After that, we optimize the distribution of the sampling points using the quasi-random sampling principle in the workspace. Then, we re-classify the sampling points based on the potential energy function, in order to ensure the safety of the mobile robot during movement.
(2) To improve the density of random points in the narrow channel, each obstacle point is first subjected to a random force and extended from eight directions. Then, the free points in the narrow channel are determined by combining the characteristics of narrow passages with the potential function.
(3) To reduce the time required for path planning, we sample the bidirectional A*, instead of the typical local path planning planner in the learning process of the traditional PRM algorithm.
(4) To shorten the distance of the path and ensure that distance from obstacles is maintained, the basic path obtained by the query process requires re-optimization by pruning techniques and potential functions, in order to improve the superiority of the final path.

The remainder of this paper is structured as follows: the Section 2 provides a the specific review of the PRM algorithm in the trajectory planning of mobile platforms in workspaces. In the Section 3, the specific combination of the APF algorithm and PRM algorithm is described in detail, including three kinds of classification of space points, the method of moving obstacle points to a narrow environment, and the optimization of local motion line segments. The Section 4 describes the optimization of the local planner used in the query process, and illustrates the specific steps for optimizing the final path in detail. Meanwhile, the principle for combining the path pruning technology with the artificial potential is described. The Section 5 details various experiments to prove that the new sampling method can effectively improve the density of free points in narrow channels. Furthermore, it is shown that the new PRM algorithm can effectively shorten the required time and reduce the length of the final path. The Section 6 summarizes the paper and describes specific future research directions.

## 2. Typical PRM Algorithm

The typical PRM algorithm is a well-known global path planning algorithm that can ensure that a robot finds a collision-free path from its specified initial position to a corresponding final position. In the typical PRM algorithm, the description of the free space is directly described by free points and collision-free line segments, which can effectively reduce the occupation of the workspace for path planning. Meanwhile, the robot can input the offline environment map for pre-processing before the actual work, in order to reduce the required time and improve the map construction efficiency. Therefore, the PRM algorithm has been successfully applied to the actual path planning problem of complex environments. The PRM algorithm is a multi-query random sampling algorithm which includes two fundamental processes: the learning process and the query process. These aspects must be analyzed and described, in order to improve the performance of the typical PRM algorithm.

### 2.1. The Learning Process

The primary task of the learning process is to construct a free space in the workspace through sampling techniques. The actual workspace of the mobile robot is denoted as $C_{s}$, which generally includes: the obstacle space, denoted by $C_{obs}$, and the free space, denoted by $C_{free}$. In the typical PRM algorithm, the free space is constructed by the learning process and can be expressed as $C_{free}=\left[nodes,edges\right]$, where the nodes represents the sampling points in the free space without collision, which are also called free points, and the edges represents the connection lines in the space without collision with obstacles.

**Figure 1:**
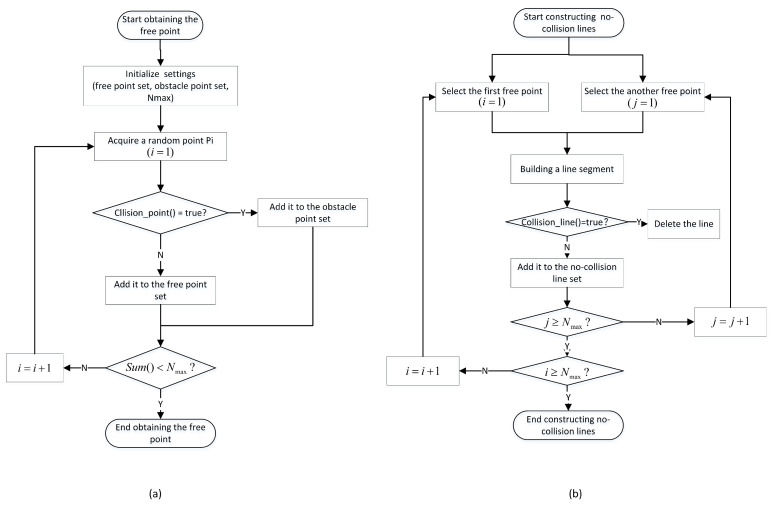
The learning process of typical PRM algorithm: (**a**) Obtaining free points; and (**b**) constructing collision-free line segments.

To realize the essential functions in the learning process, the specific steps for obtaining free points are shown in Figure 1a. First, we need to initialize the critical parameters for obtaining the free point set, including: setting the free point sets and the obstacle set in the free space as empty, setting the maximum number of free points as $N_{max}$, and adding the initial and final positions into the free point set. Next, we choose a sampling point in the environment using the random sampling principle and set the index of the random point as 1 (i=1). Then, the random point is submitted to the collision detection function. If it does not collide, the point is added to the free point set; otherwise, the point is put into the obstacle point set. Finally, we judge whether the sampling task is finished. When the value is smaller than the number of the free point set, we continue the sampling task, setting the index as (i=i+1); otherwise, the sampling task is complete.

While describing the free space using the free point set, the number and the distribution state of the sampling points may seriously affect the final free space of the workspace. Due to the slow development of sampling principles, researchers have mainly obtained sampling points using random sampling principle, ensuring that the distribution of sampling points covers the whole workspace by increasing the number of random points. After completing the description of the free space using the free point set, we directly use collision-free line segments between free points to represent effective local paths. These collision-free lines can ensure that the robot can perform motion in free space.

The specific steps of building the collision-free line segments are shown in Figure 1b. First, we select the first free point $P_{i}\left(i=1\right)$ and another free point $P_{j}\left(j=1\right)$ in the free space point set, in order to construct a line segment. Next, the line segment is submitted to collision detection to build the collision-free line. When a collision occurs along the line, we directly discard the line segment and continue to another sampling point $P_{j}\left(j=j+1\right)$; otherwise, we add the two endpoints of the path to the edge set. Then, we judge whether the point $\left(P_{j}\right)$ is the end point of the free point set. If the value of *j* is less than $N_{max}$, we assign the random sampling index to j=j+1 and continue to construct a new line segment, based on another free point $P_{j}$; otherwise, the point indicates the endpoint of the free point set. Finally, we judge whether the point $\left(P_{i}\right)$ is the end point of the free point set. If the value of *i* is less than $N_{max}$, we assign the random sampling index to i=i+1, and continue to construct a new line segment based on another free point $P_{i}$; otherwise, this point indicates the endpoint of the free point set, and the free line segment sampling is complete.

When the number of the free points in free space is determined, the efficiency of collision detection and the total number of line segments may seriously affect the construction time of free line segments. To shorten the required time for collision detection, researchers haved used the binary collision detection methods, instead of the traditional incremental collision detection method. Meanwhile, researchers have also used local line segment connections, instead of overall line segment connections, in order to reduce the number of overall free line segments and the impact on path construction. Through optimization in these two aspects, the required time for constructing the free line segments can be significantly shortened, compared to the typical learning process.

### 2.2. The Query Process

The primary purpose of the query process in the PRM algorithm is to use local path planning to find an effective path by querying the collision-free paths constructed in the learning process, thus obtaining a practical path from the initial position to the final position. As the collision-free local path is constructed through the learning process, the robot does not need to build local collision-free paths in the entire free space. The performance of the local path planner affects the time required for the query process in the PRM algorithm. At present, the principal local path planners in the query process include the A* and D* algorithms. Compared with the D* algorithm, the A* algorithm adds a heuristic function to determine the optimal path, and has higher efficiency for path planning in static workspaces. Therefore, the A* algorithm has become the mainstream local path planner in the query process of the typical PRM algorithm. In completing the query process, we need to evaluate the basic performance of each free point using the typical A* algorithm, which is expressed as:

(1) $$f\left(x\right)=g\left(x\right)+h\left(x\right)\;$$

where f(x) represents the performance value of the specified free point; g(x) is the operating cost function, which represents the Euclidean distance (actual cost) from the initial position to the specified position; and h(x) is the Heuristic function representing the distance from the current position to the final position.

**Figure 2:**
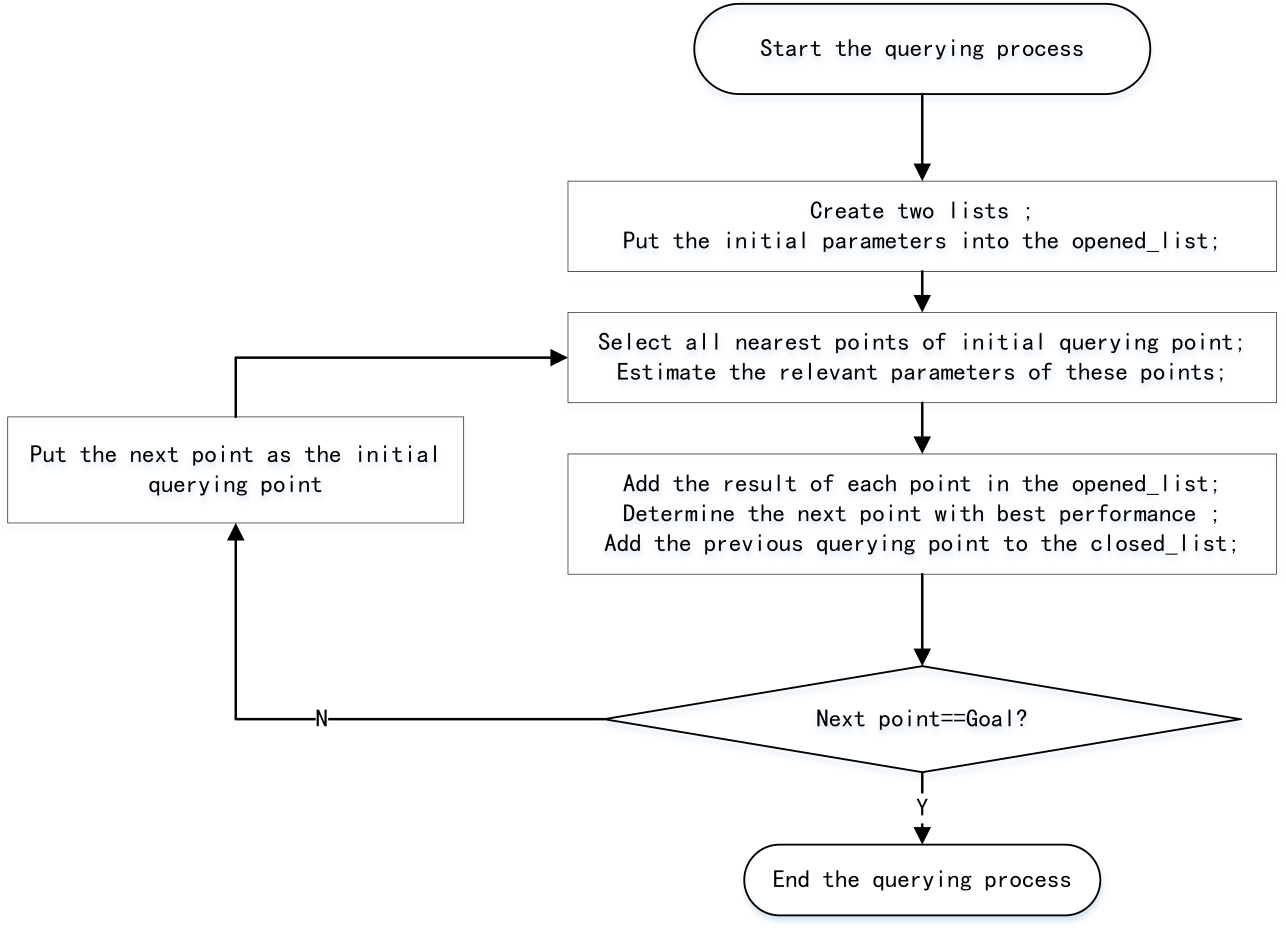
The querying process of typical PRM algorithm.

We can effectively determine the collision-free trajectory using the A* algorithm by adding the initial and target points to the map. The specific steps of the querying process in the typical PRM algorithm are shown in the Figure 2. First, we create the opened-loop list and closed-loop list to save the relevant parameters. The closed-loop list is used to store the free point that will not be considered again, but the opened-loop list is used to store the whole free point considered to find the shortest path. We also assigned the initial parameters to the opened-loop list, including the index of the initial point, the performance parameters, and the number of closed points. Next, we find the overall nearest free points that can form a local path with the initial query point, and estimate the relevant performance parameters of these nearest free points. Then, we select the free point $\left(S_{1}\right)$ with the smallest sum of cost functions as the the nearest point with the best performance, and delete the previous query point from the open-loop list. Finally, we check whether the nearest point $\left(S_{1}\right)$ has reached the target point; if it does not reach the target point, we add the previous point into the closed-loop list, set the query point as the nearest node $\left(S_{1}\right)$, and continue to query the next free point with the best performance; otherwise, we have found the global path and the querying process is complete.

Path planning for a mobile robot in a complex environment can be realized through the above learning and query processes. However, finding a collision-free motion path in a narrow passage may be impossible, due to the randomness of the free points in the learning process. However, when the number of sampling points is increased, the sampling time and the number of sampling points may inevitably be wasted. To ensure the mobile robot’s safety in the actual movement, we hope that there is a certain distance between the robot and the obstacle to avoid collision. In this paper, we propose an optimized PRM path planning algorithm by effectively combining the artificial potential energy function, in order to solve the above problems.

## 3. The Optimized Learning Process

**Figure 3:**
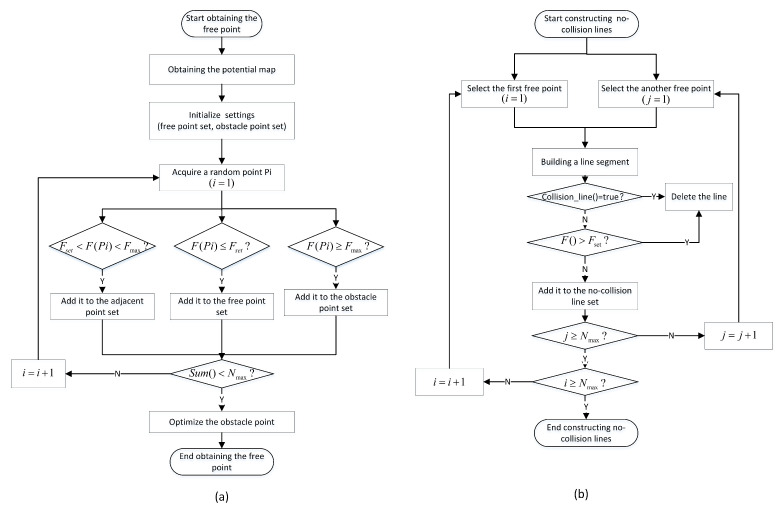
The learning process of the optimized PRM algorithm.(**a**) Obtaining free points; and (**b**) constructing collision-free line segments based on the optimized PRM algorithm.

This section mainly details the optimization of the learning process to obtain free points in complex environments, especially inside narrow passages. Then, we improve the collision-free line segment in free space, in order to make it more suitable for the motion performance of the mobile robot. The optimization content of the learning process mainly includes re-defining the initial sampling point, optimizing the obstacle point, and improving the collision-free line segment. The specific steps of sample point acquisition and collision-free path construction in the learning process are shown in Figure 3.

### 3.1. Optiming the Workspace Map and the Initial Point Classification

To complete the path planning task, the traditional algorithm first simplifies the environment into a typical two-dimensional map. To ensure the safety of the mobile robot, the traditional method expands the obstacle space according to the diagonal length of the mobile platform and the safe distance during movement. We used the typical PRM algorithm to obtain the free workspace, which is mainly composed of free points and collision-free line segments, where the free points serve as the basis for forming the collision-free line segments. However, when facing a complex environment with various narrow channels, the above obstacle expansion methods may lead to path planning failure, as the maximum width of the narrow channel may become smaller with the expansion of obstacles. Therefore, the difficulty of obtaining free points in narrow channels based on random sampling methods is increased. For narrow passages that are only suitable for mobile robots, the above obstacle expansion method leads to connections between obstacles. Therefore, some narrow channels cannot be described in the map, resulting in the path planning failure in the complex environment with narrow channels. Therefore, the obstacle expansion method must be optimized to display all narrow channels suitable for traversal by the mobile robot.

Considering the path planning problem in a complex environment with narrow channels, we first re-define the obstacle expansion method in the work environment to retain all narrow channels suitable for the mobile robot. Then, we replace the traditional random sampling technology with typical standard sampling techniques, in order to enhance the distribution of the sampling points. Finally, we decompose the sampling points into three categories, thus reducing the number of the free point, which can ensure the safety performance of the final acquisition path.

(1) Re-define the workspace map

In traditional path planning algorithms, researchers generally deal with obstacle expansion according to the safety distance and the diagonal length of the mobile robot when the environment for the mobile robot does not include various narrow channels. For complex environments with various narrow channels, some narrow channels suitable for mobile robot may disappear in the map, especially those that just meet the movement conditions of the mobile robot. This is caused by the coincidence of the data of obstacles during map zoom-out or the obstacle expansion process. In order to obtain all narrow channels for the mobile robot, we must perform the obstacle expansion using the diagonal parameters of the mobile platform. With this method, some narrow channels can assigned as suitable for the mobile robot, while the rest are deemed as not suitable for its motion. We can retain and display all narrow passages where the mobile robot will not collide with obstacles, even if the channel is just large enough to place the mobile platform in stationary state. Therefore, we first directly expand the obstacles based on the diagonal length of the mobile robot, ignoring the movement safety distance.

Through the above method, we can simply obtain the complex working environment as a two-dimensional map with narrow channels. To ensure the safety of the mobile robot located at the sampling point, we then re-classify the sampling points based on the safe distance limit during movement. Meanwhile, we add safe distance constraints to construct the collision-free line segments and optimize the querying process.

(2) Enhance the distribution of the sampling points

After obtaining the typical two-dimensional map, it is necessary to conduct multi-point sampling in the environment through use of random sampling principle, for which it is necessary to determine the type of sampling points. As a typical spatial sampling technique, the random sampling method has been widely used for the PRM map construction step. This method can realize effective sampling of multi-dimensional environments. However, due to the randomness of the sampling points, it is necessary to set a higher sampling number to ensure full-space sampling, which can waste time and reduce the efficiency of the PRM algorithm. Meanwhile, the random sampling method defined by Monte Carlo method cannot be applied to the sampling of complex environments with narrow channels. A low number of sampling points cannot effectively distribute multiple free points in the channel, while a high number of sampling points can increase the density of free points, resulting in concentrated free points in free space. Therefore, we must enhance the sampling point distribution to adequately realize sampling of the whole working environment.

To improve the uniformity of the sampling points over the whole space, we adopt the quasi-random sampling principle, instead of the typical random sampling principle, for the free space. The quasi-random sampling principle defined by the Monte Carlo method is currently mainly used to solve global optimization problems, which considers the discrete degree of the random point distribution. This new method has been shown to possess the same efficiency as the typical random sampling method. The sampling points based on the quasi-random sampling principle are also random, but the dispersion degree is higher in the spatial point set. Halton sets are some of the most typical point sets obtained by quasi-random sampling. Once the number of random sampling points is set, this method can ensure the uniform distribution of all sampling points throughout the entire workspace, which means that all sampling points are uniformly distributed inside the whole working environment. In the specified workspace, the sampling point set can be obtained by the Halton sets defined by the quasi-random sampling principle, as has been shown [35]. In brief, we choose *d* distinct primes $P_{1},P_{2},\cdots ,P_{d}$, and the set’s $i^{\mathrm{th}}$ point is given by:

(2) $$P_{i}=\left[r_{p_{1}}\left(i\right),r_{p_{2}}\left(i\right),\cdots ,r_{p_{d}}\left(i\right)\right],i=0,1,\cdots n-1,\;$$

where $P_{i}$ represents the sampling points in the sampling space and $r_{p}\left(i\right)$ is obtained by writing the digits of the *p*-ary notation for *i* in reverse order, which can be expressed explicitly as:

(3) $$\left\{\begin{array}{c} r_{p}\left(i\right)=\frac{a_{0}}{p}+\frac{a_{1}}{p^{2}}+\frac{a_{2}}{p^{3}}+\cdots \\ i=a_{0}+pa_{1}+p^{2}a_{2}+\cdots ;a_{j}\in \left\{0,1,\cdots p-1\right\} \end{array}\right.,$$

where *i* is a real number written in *p*-ary notation.

**Figure 4:**
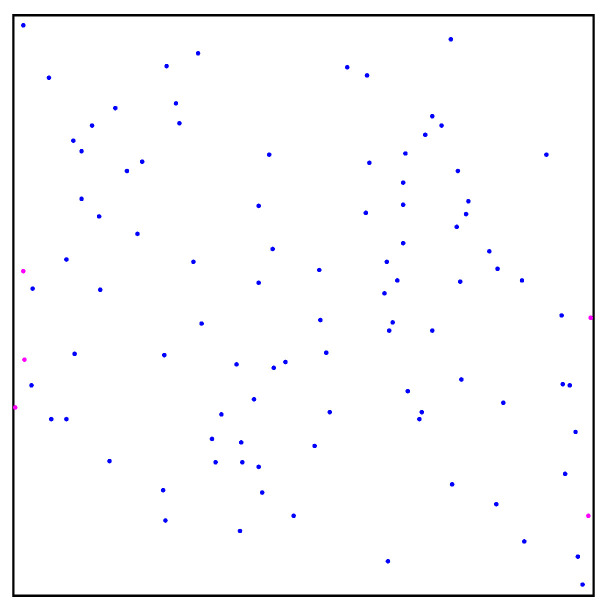
Sampling points obtained by random sampling principle.

**Figure 5:**
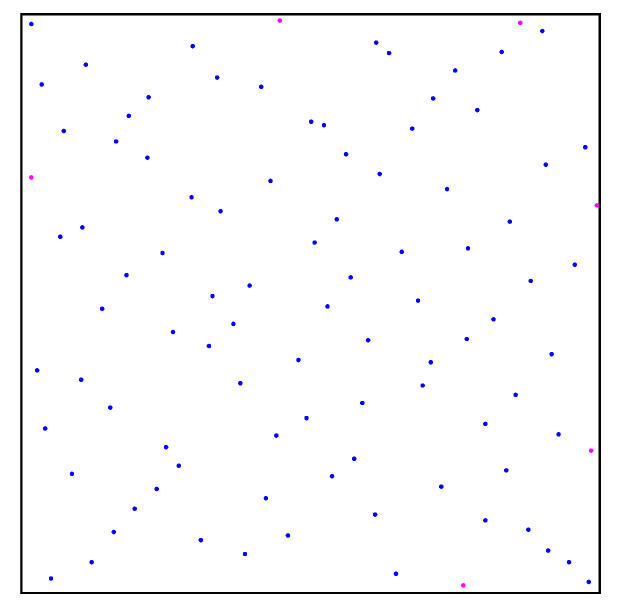
Sampling points obtained by quasi-random sampling principle.

We set the number of sampling points to 100 and carried out random sampling in the specified workspace using the two methods. The sampling results are shown in Figure 4. By comparing the different distribution results of random points, we found that: (1) Some random points obtained by the random sampling principle in the free space have a shorter distance than the ordinary distance between sampling points based on the quasi-random sampling principle, as shown in Figure 4 and Figure 5. Therefore, randomly sampled points based on the random sampling principle may be concentrated together, as can be seen from Figure 4. (2) Due to the randomness of the random sampling principle, some free spaces without any sampling points occur, as shown in Figure 4, while areas surrounded by sampling points do not have a large space, as shown in Figure 5. Therefore, the quasi-random sampling principle can effectively reduce the concentration of random points, compared with the typical random sampling method. The optimized method evenly distributes all the sampling points in the overall space, in order to improve the efficiency of the learning process.

(3) Re-classify the sampling points

**Figure 6:**
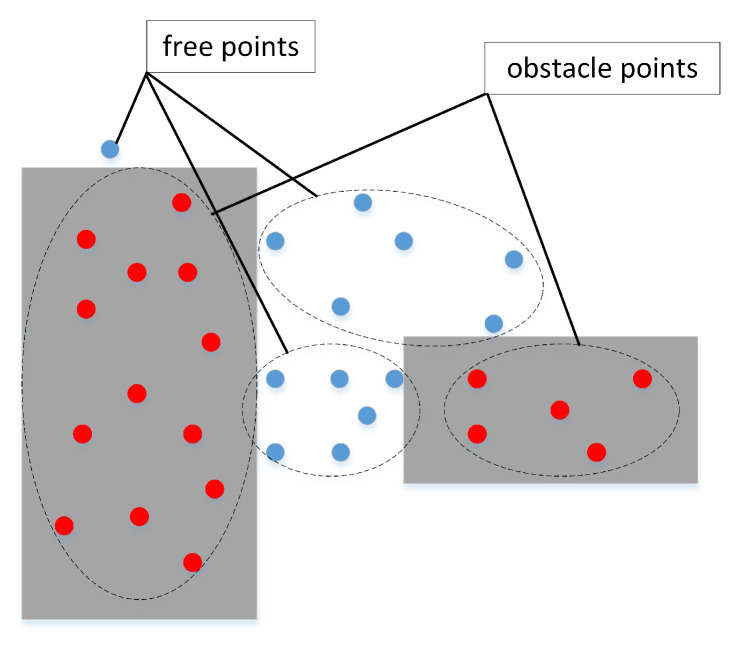
Typical classification of sampling points.

**Figure 7:**
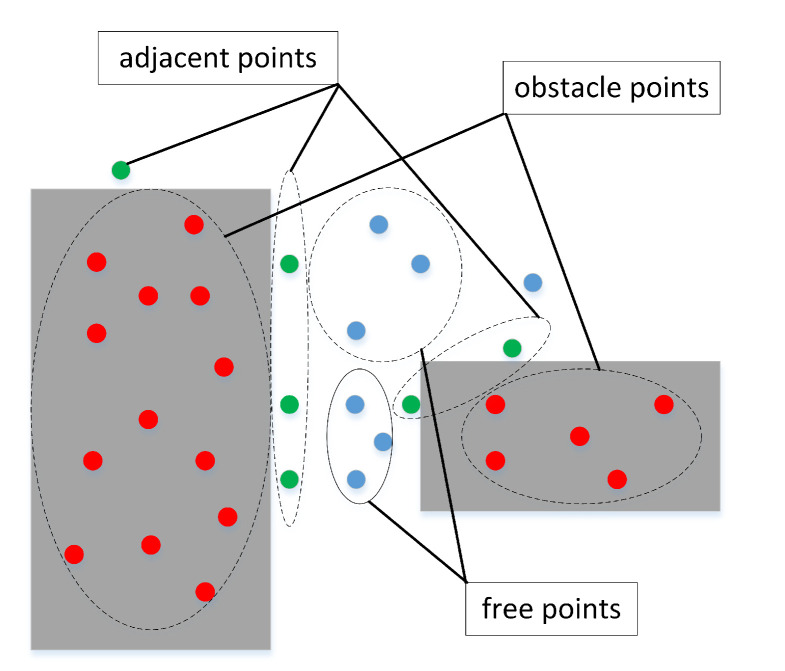
Optimized classification of sampling points.

Through the new obstacle expansion technology, we obtain a variety of narrow channels that can hold the mobile robot at rest. To meet the actual movement requirements, we need to take the safe distance of the robot’s movement into account in the path planning process. To avoid collisions between the mobile robot and the obstacles during movement, we optimize the free points obtained by the quasi-random sampling principle. Considering the safety distance during actual movement, we decompose the traditional sampling points into three categories: free points, adjacent points, and obstacle points. The obstacle point set is the same as the typically defined one, meaning that the sampling points fall into the set of random points in the obstacle space. However, the free point set not only needs to fall into the free space, but also requires the shortest distance between these points and the nearest obstacle to be greater than a specified distance. Then, the adjacent point set is the rest of the random points in the free space, which represents random points in free space whose shortest distance to the obstacle is less than the specified distance. Comparing the traditional classification method in Figure 6 and the optimized classification method in Figure 7, the adjacent points cannot guarantee safe distance limits between the mobile robot and obstacles. Therefore, the adjacent points need to be discarded, and cannot be used to construct the collision-free line segments.

The three different point sets proposed in this paper are not only related to the distribution in free space, but also to the closest distance to obstacles. According to the distance constraint between the obstacle and the sampling point, we use the artificial potential energy function to accurately classify the sampling points, which requires the analysis and description of the repulsive force generated by obstacles in the workspace. The artificial potential field is typically used to construct an effective map and find the shortest path, according to the natural laws of attraction and repulsion. However, the artificial potential field in this paper mainly optimizes the critical steps in the PRM algorithm. In the sampling process, we use only the repulsive function alone to construct the repulsive force field, in order to classify all sampling points. For any specified workspace, we can construct a potential energy map based on the basic information of the map and the potential function.

Let a random sampling point in the free space be represented as $P_{o}$, and the $i^{\mathrm{th}}$ obstacle point be expressed as $P_{osi}$. Then, the distance between the two points is:

(4) $$d_{ri}=∥p_{o}-p_{osi}∥=\sqrt{{\left(x_{o}-x_{osi}\right)}^{2}+{\left(y_{o}-y_{osi}\right)}^{2}},\;$$

where $\left(x_{o},y_{o}\right)$ is the position of the random point in the two-dimensional environment, $\left(x_{osi},y_{osi}\right)$ represents the position of the $i^{\mathrm{th}}$ obstacle, and *d* represents the nearest straight-line distance from the random point to the obstacle.

In the potential function, the repulsive force will acts only on the nearby points around the obstacle. Therefore, the repulsive force on the points far away from the obstacle’s position is equal to zero. The repulsive force at any point in the space can be specifically expressed as:

(5) $$F\left(p_{o}\right)=\left\{\begin{array}{cc} \frac{1}{2}k_{ri}\left(\frac{1}{d_{ri}}-\frac{1}{d_{set}}\right), & d_{ri}\leq d_{set};\\ 0, & d_{ri}>d_{set}; \end{array}\right.$$

where $k_{ri}$ represents the repulsion scale factor, $d_{ri}$ represents the minimum distance between the point and the $i^{\mathrm{th}}$ obstacle, and $d_{set}$ represents the influence distance of each obstacle.

**Figure 8:**
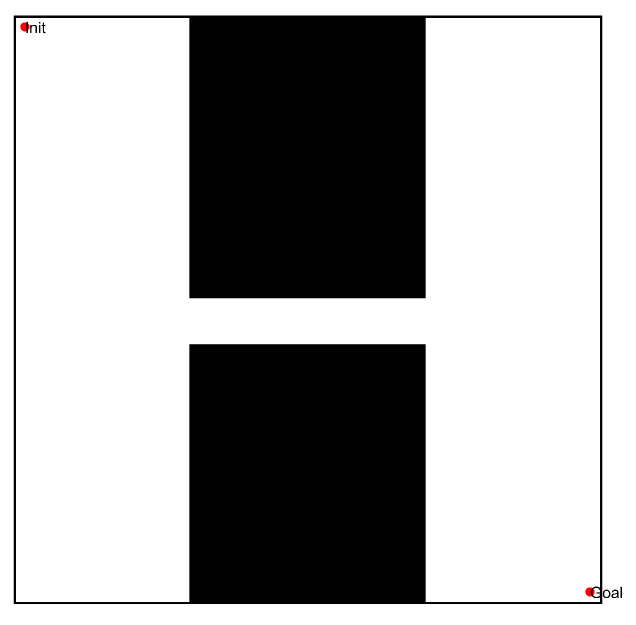
A typical working environment.

**Figure 9:**
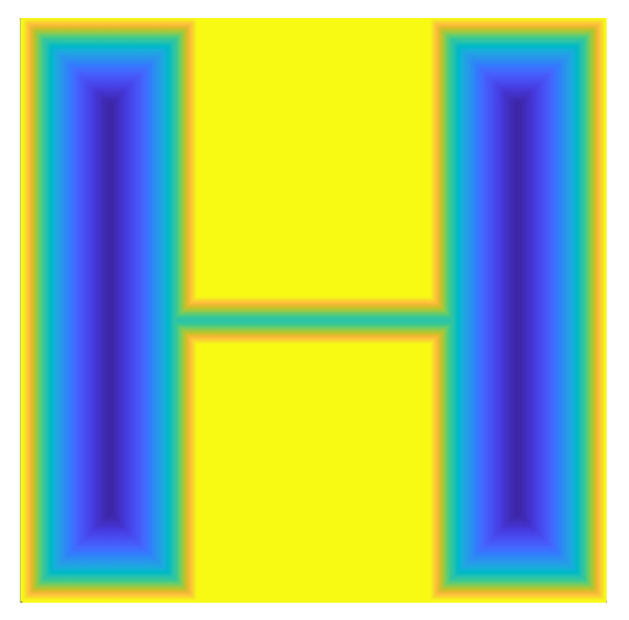
Potential energy map.

For the workspace with obstacles shown in Figure 8, a corresponding potential map containing only contained repulsive forces can be established through the above potential energy function, as shown in Figure 9. Once the points in the free space are far away from the obstacle (greater than $d_{min}$), the value of the repulsive force is zero, based on the potential map in this figure. In a specified field, the repulsive force increases with decreasing distance from the obstacle. When the point falls on the boundary or the sounding of the obstacle, the repulsive force is at its largest. Therefore, using the value range of the potential energy function can effectively enhance the classification of all sampling points.

After creating a three-dimensional potential map using the artificial potential function, we can use the quasi-random sampling principle to obtain random sampling points from the workspace. These sampling points can then be decomposed into three different sets, according to the specified potential force, which can be expressed as:

(6) $$\left\{\begin{array}{c} S_{f}=\{{p_{f}}_{i}\left|\right|{p_{f}}_{i}\in S,F\left(x_{fi},y_{fi}\right)<F_{set}\}\\ S_{n}=\{{p_{n}}_{i}\left|\right|{p_{n}}_{i}\in S,F_{set}\leq F\left(x_{ni},y_{ni}\right)<F_{\max}\}\\ S_{o}=\{{p_{o}}_{i}\left|\right|p_{on}\in S,F\left(x_{oi},y_{oi}\right)<F_{\max}\} \end{array}\right.$$

where ${p_{f}}_{i}$, ${p_{n}}_{i}$, and ${p_{o}}_{i}$ represent free points, adjacent points, and obstacle points, respectively; $S_{f}$, $S_{n}$, and $S_{o}$ represent the set of free points, the set of adjacent points, and the set of obstacle points, respectively; F() represents the repulsive force based on the artificial potential energy function; and $F_{\max}$ represents the maximum repulsive force generated by the specified distance.

For any specified workspace, we can obtain three different point sets by combining the quasi-random sampling principle and the optimized classification technique proposed in this paper. The solution process for the the optimized classification technique is illustrated in Figure 3a. First, we use the artificial potential function to solve the repulsive force and then construct the three-dimensional potential energy map based on the obtained repulsive force. Next, we determine the three different point sets and the total number of overall sampling point sets. Then, we obtain one sampling point, based on the quasi-random sampling principle, and estimate the repulsive force at the sampling point. Finally, the corresponding point is classified into the specific point set, based on the potential function, and we determine the total number of sampling points. If it is less than the initial setting number, we continue to acquire new sampling points; otherwise, the sampling process is complete.

**Figure 10:**
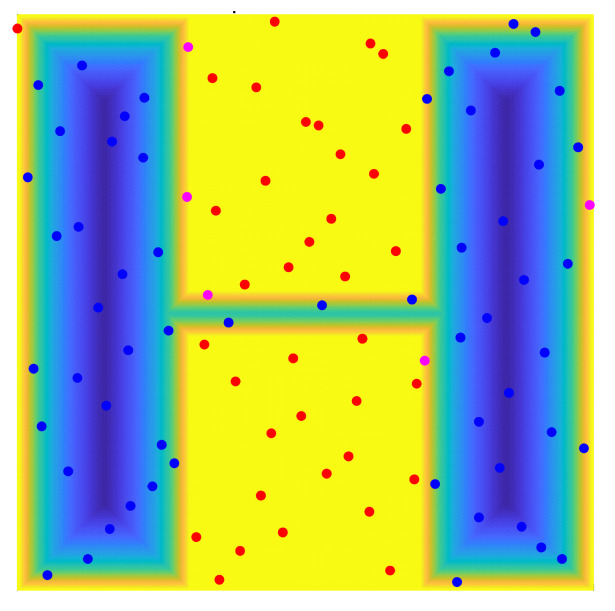
The distribution of sampling points in the potential energy map.

**Figure 11:**
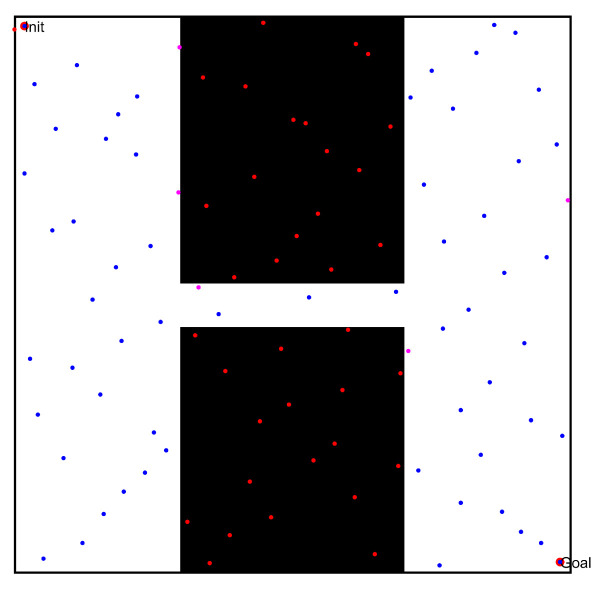
The distribution of sampling points in the working environment.

For the workspace shown in Figure 8, the above method was used to construct free space and obstacle space through the three different sampling point sets, and the specific results are shown in Figure 10. In this figure, blue points represent free space points, pink points represent adjacent space points, and red points represent obstacle points. Among these three types of sampling points, free points can be used to construct free space, obstacle points can become sampling points in narrow passages by optimizing these obstacle points (as proposed in the next subsection), and adjacent points are closer to obstacles only can be abandoned. We add these three types of random points into the two-dimensional workspace to directly observe the distribution of these sampling points. Figure 11 specifically shows the classification results of these random points in a two-dimensional working environment.

### 3.2. Optimizing the Obstacle Points by Artificial Potential Field

In the typical PRM algorithm, the obstacle points are generally discarded directly, and the number of sampling points in the narrow channel is increased by increasing the number of total sampling points. In the optimized PRM algorithm based on Gaussian sampling, the obstacle points are moved to the surroundings of the obstacle by adding disturbance, inevitably increasing the number of free points in the narrow channel. Another optimized PRM algorithm, based on bridge sampling, directly adds the midpoint of the two obstacle points collected by different obstacles to the narrow channel. In the sampling processing of the narrow channel, the above methods must perform sampling multiple times, while the number of sampling points in the narrow channel cannot be directly increased. In this paper, the obstacle points are directly optimized by combining the characteristics of obstacle points and narrow passages, thereby effectively increasing the sampling number of free points in the narrow channels.

**Figure 12:**
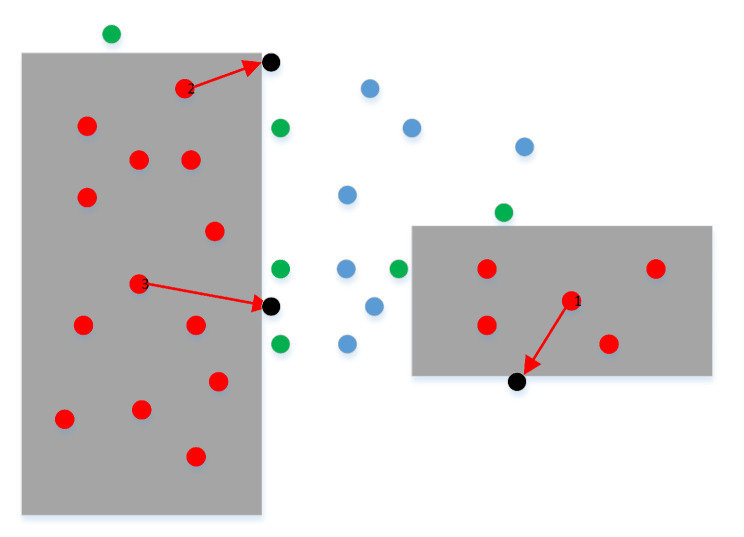
Optimizing obstacle points as the free points in free space.

To optimize the obstacle points, we need to directly move the obstacle points into the free space to become typical free points. Meanwhile, we can effectively transform the obstacle points into adjacent points through the effective combination of the force of the random point in space and the specified minimum repulsion force. First, we set a random force on the obstacle point to determine the initial movement direction, as shown for point 1 in Figure 12. Second, the obstacle point is required to move, gradually increasing its distance according to the initial force direction. Finally, the optimized obstacle point can be obtained by comparing the minimum repulsive force for determining adjacent points and the repulsive force of the obstacle point moving along the force direction. Therefore, the repulsive force and the movement distance of the obstacle point can be expressed as:

(7) $$\begin{array}{cc} \left\{\begin{array}{c} F_{S}=F_{0}+d*\vec{F}\\ \vec{F}=rand\left(1,2\right)\\ d=\min(num) \end{array};\right. & if(F\left(P_{S}\right)<F_{set},num=1,2,\cdots ,N) \end{array}$$

where $\vec{F}$ represents the direction of the random force on the obstacle point, $F_{0}$ represents the initial repulsive force of the obstacle point, $F_{S}$ represents the repulsive force after moving some distance, and *d* represents the moving distance of the obstacle point.

The obstacle point can be guaranteed to become a free point after moving to free space under a random force; however, the free point may be transformed into a narrow passage or a free space far away from the obstacles. To increase the density of sampling points in the narrow channel, we must actually guarantee that the free point falls in the narrow channel. As a narrow passage is composed of at least two obstacles, the obstacle point will inevitably collide twice during movement in the narrow passage. Similarly, two numerical transformations of the repulsive force must occur, compared to the specified repulsive force during movement along the direction of the virtual repulsive force. Based on the characteristics of narrow passages, the corresponding positions determined by the numerical transformations must be in the narrow passage; otherwise, they are outside the narrow passage. The specific sampling results are shown in Figure 12. The specific constraints for adding these free points in a narrow passage can be expressed as follows:

(8) $$\left\{\begin{array}{c} P_{1}=\left\{p_{i}^{1}|f\left(p_{i}^{1}\right)<F_{set}\cap f\left(p_{i-1}^{1}\right)>F_{set}\right\}\\ p_{i}^{1}=P_{x}+d(i+1)\cdot dir_{f};\left(i=1,2,...n_{1}\right)\\ P_{2}=\left\{p_{j}^{2}|f\left(p_{j}^{2}\right)<F_{set}\cap f\left(p_{j+1}^{2}\right)>F_{set}\right\}\\ p_{j}^{2}=P_{x}+d(j+1)\cdot dir_{f};\left(j=n_{1},n_{1}+1,...\right) \end{array}\right.$$

where f() represents the repulsive force at any point, $P_{1}$ represents the first adjacent point, $P_{2}$ represents the second adjacent point, *d* represents the moving distance, and $F_{set}$ indicates the maximum repulsive force.

**Figure 13:**
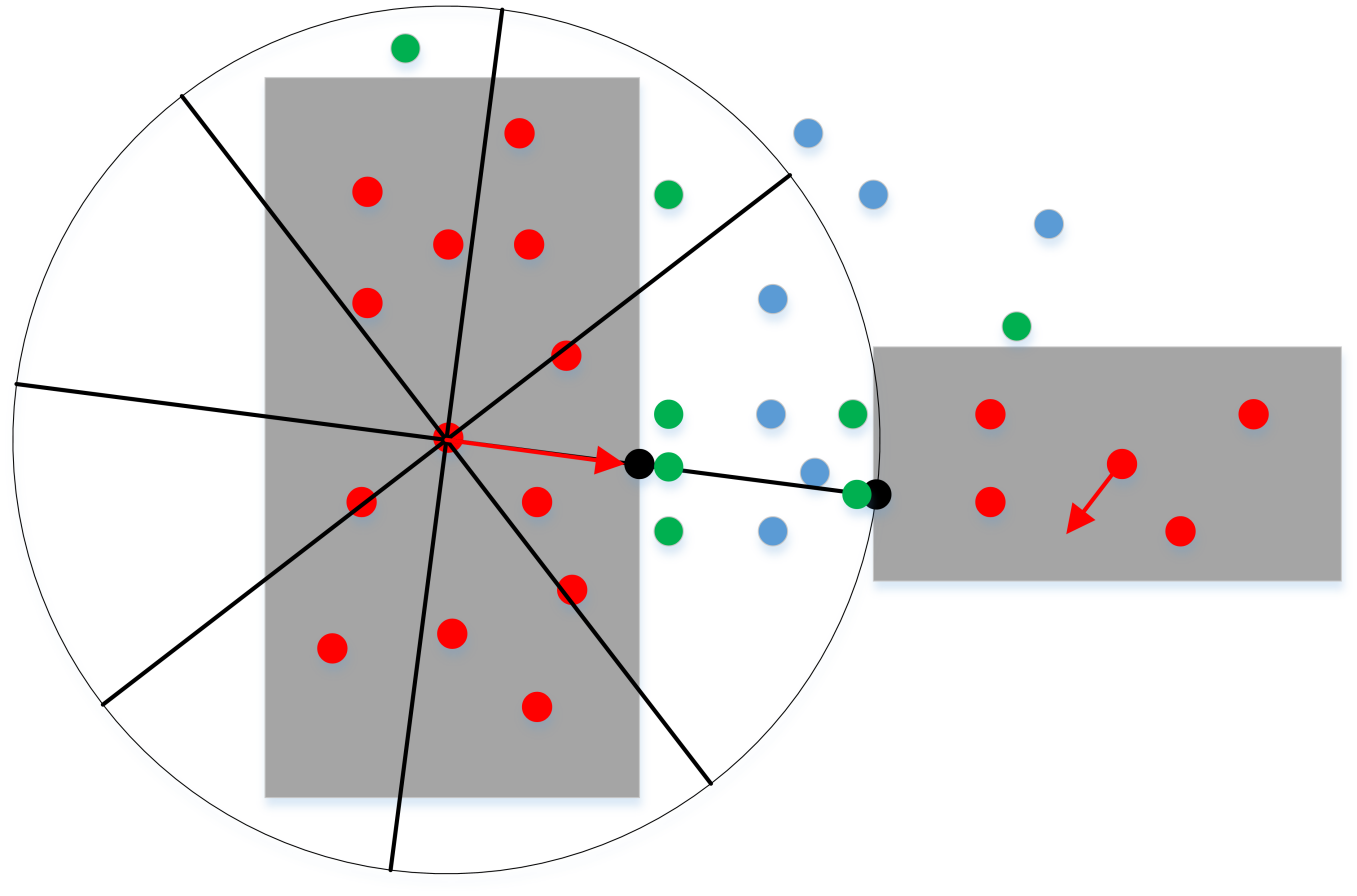
Optimizing obstacle points as the free points in narrow passages.

An obstacle point optimized by direct movement cannot be guaranteed to transform into an adjacent point, due to the uncertainty of the random force; for example, see Figure 12. To improve the maximum possible points falling into the narrow passage, we continue to add other motion directions, different from the basic direction of the random force. Then, the obstacle point performs the motion transformation in these directions at the same time. Finally, we select the direction that first occurred in these two numerical transformations as the best motion direction, and regard the transformation points as two results after optimizing the obstacle point. The specific solution results are shown in Figure 13.

According to the basic principle detailed above, the critical technology of obstacle point optimization lies in the determination of the random force direction and the change in repulsive force of the obstacle point moving along the eight directions. After acquiring the obstacle point set, we can count the total number of obstacle points and optimize each point as free points in narrow channels. The specific solution process for optimizing each obstacle point is shown in Algorithm 1. In the process, we first assign an random force to the obstacle point and re-assign a random point whose force is zero. Next, we solve the actual angle of the random force and solve in all eight directions, according to the initial angle of the random force. Finally, we expand the obstacle point in eight directions gradually, and count the state transformation between the repulsive force of the expansion point and the specified repulsive force. When two state transformations occur first, we take the corresponding two positions as the optimization result of the obstacle point.
**Algorithm 1** Res_Optm = Optimize_Point_Obs(Point,Force_rand,Rep_Force,map).1:**if** Force_rand==0 **then**2:    F_rand = rand(1,2);3:**end if**4:Force_dir = atan2(Force_rand(1),Force_rand(2));5:**for** i = 1:1:8 **do**6:    dir(i,:) = Extend_Force_dir(Force_dir);7:**end for**8:[Point_Opt1;Point_Opt2] = Obtain_Point(Point,Rep_Force,map);9:Res_Optm = [Point_Opt1;Point_Opt2];10:Return Res_Optm

**Figure 14:**
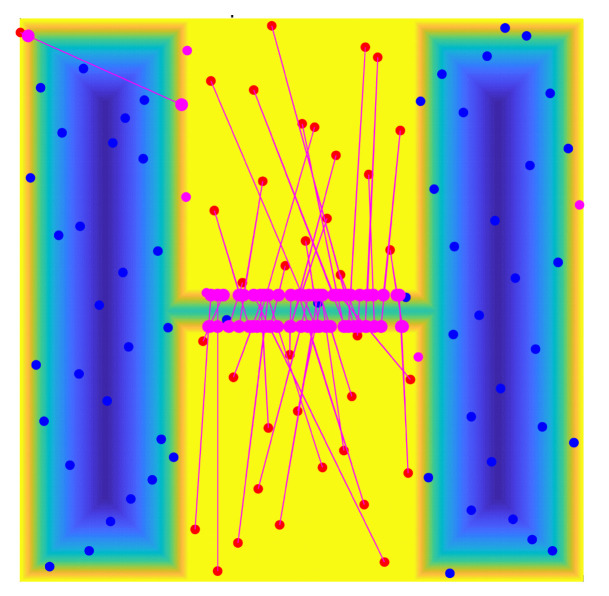
Optimization results of the obstacle points in the potential map.

**Figure 15:**
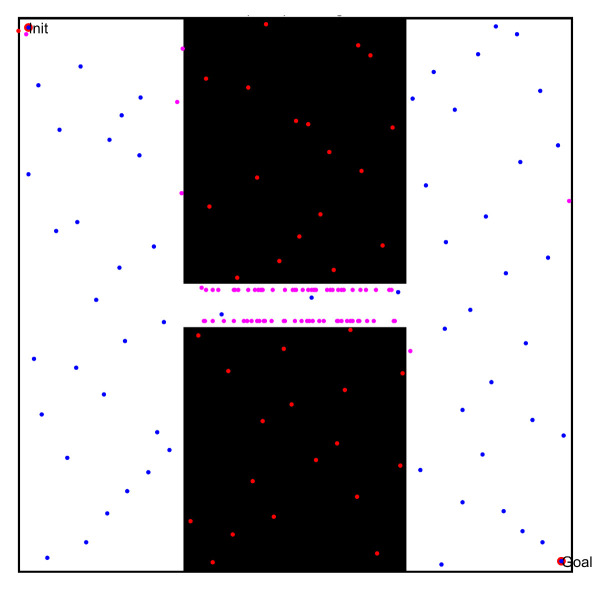
Optimization results of the obstacle points in the working environment.

In the potential map based on the above workspace, we used the above obstacle point optimization technology to move all obstacle points into the free point set; the specific results are shown in Figure 14. According to the distribution of the sampling points, we can see that (1) most obstacle points were effectively moved from the obstacle space to the narrow channel, effectively increasing the density of the sampling points in the narrow channel, and (2) some obstacle points may be misjudged when the obstacle points fall in the surroundings of the workspace, because the boundary of the workspace is set as an obstacle. However, the probability of sampling in the surrounding boundary by the quasi-random sampling method is low. Therefore, the above phenomenon occurred only for a few obstacle points, thus having a low impact on the optimization of all obstacle points. The optimized results of all obstacle points were mapped into the actual workspace to directly observe the free point distribution results, as shown in Figure 15. According to the distribution of sampling points in the narrow channel before and after the optimization of the obstacle point set, it can be seen that the obstacle point optimization technology proposed in this paper can significantly improve the sampling density of free points in the narrow passage.

### 3.3. Improving Collision-Free Line Segments Using the Repulsive Force

After adopting the quasi-random sampling method and obstacle point optimization processing, the density of sampling points in the narrow passage was significantly increased, and the distribution of sampling points in the free space did not appear to be concentrated. Next, we performed connection processing, based on the above-mentioned free point set. This process aims to construct the local path through line segments that do not collide with the obstacle space. Some researchers have directly connected one free point with all other points in the free space and performed collision detection to obtain line segments. Furthermore, the two free points and line segments that do not collide may be used together to build a free space. In this process, the number of line segments is large after connecting all points, and the local path needs to be segmented during collision detection. Therefore, the number of paths and the length of the total paths will inevitably lead to a high time being required to construct the free space. To date, researchers have reduced the number of overall segments by designing a specified range for one free point for connection processing and improving the collision detection performance by adopting the binary collision detection technique, instead of the incremental collision detection technique.

In the actual construction of line segments, the connection state in the large free space is less complex than the connection state in the narrow passage. It is easy to collide with the surrounding environment when connecting the free points in the free space to free points in the narrow passage. At the same time, the mobile robot does not want to move at these positions near to the obstacle, even if it does not collide. Considering the above-mentioned actual motion requirements, we propose a new type of connection state, considering the three essential types of sampling points and potential energy functions, in order to avoid collision and to stay away from the obstacle space.

The sampling points obtained by the optimized sampling technology include free point sets, adjacent point sets, and obstacle point sets. In the actual line segment construction process, we need to ensure that all sampling points in the free space do not collide and that the distance between a given free point and the nearest free points is greater than a specified distance. Therefore, we can only select the free points as sampling points during the connection process, such that the adjacent and obstacle point sets need to be discarded. Meanwhile, we connect the specified free point with the other free points in the deigned specified space to construct line segments, in order to optimize the time required for connection processing. According to the repulsive function, the other free points in the deigned specified space can be specifically described as:

(9) $$\left\{\begin{array}{c} P_{o}=\left\{p_{i}|p_{i}\in S_{f}\right\}\\ P_{oi}=\left\{p_{i},\left(i=1,2,\cdots n\right)|p_{i}\in S_{f}\right\}\\ D_{oi}=\left|\right|P_{0}-P_{i}\left|\right|<d_{set} \end{array}\right.$$

where $P_{o}$ represents a free point in the free space, $p_{oi}$ represents the other free points in the deigned specified space, $d_{oi}$ represents the distance between the two free points, and $d_{set}$ represents the radius limit for the specified space.

The positions of two endpoints meet the collision-free conditions when constructing a line segment. Moreover, the line segment constructed by the two points needs to ensure that no collision with obstacles occurs, and the distance between the line segment and the nearest obstacle should be no less than the specified distance. According to the repulsion function, the distance between the line segment and the nearest obstacle needs to be limited, in order to completely avoid the possibility of collision, which can be expressed as:

(10) $$\left\{\begin{array}{c} edge=\left\{P_{o}P_{i},P_{1}P_{i},\cdots P_{i}P_{j}\right\};\left(i,j=1,2,\cdots n;i\neq j\right)\\ F\left(P_{i}\right)<F_{set};F\left(P_{j}\right)<F_{set};\max F\left(P_{i}P_{j}\right)<F_{set}\\ D_{ij}=\left|\right|P_{i}-P_{j}\left|\right|<d_{set} \end{array}\right.$$

where edge represents a free collision-free line segment in space, maxF represents the maximum repulsive force of the line segment, F() represents the repulsive force at any point, and $F_{set}$ represents the repulsion based on the shortest distance to the obstacle.

**Figure 16:**
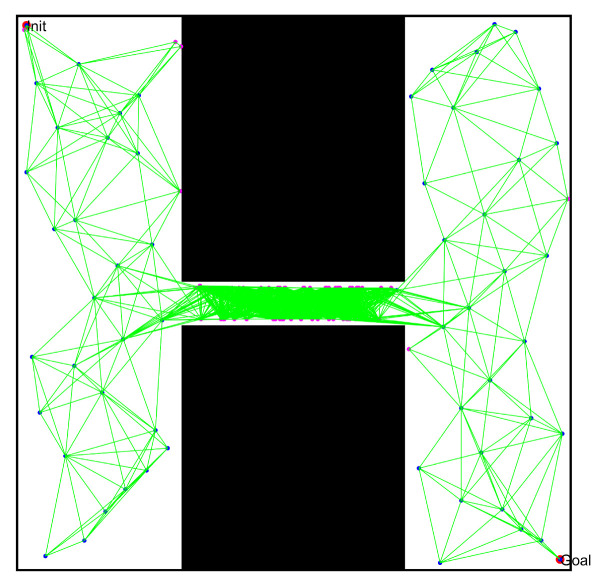
Free space construction based on the typical PRM algorithm.

**Figure 17:**
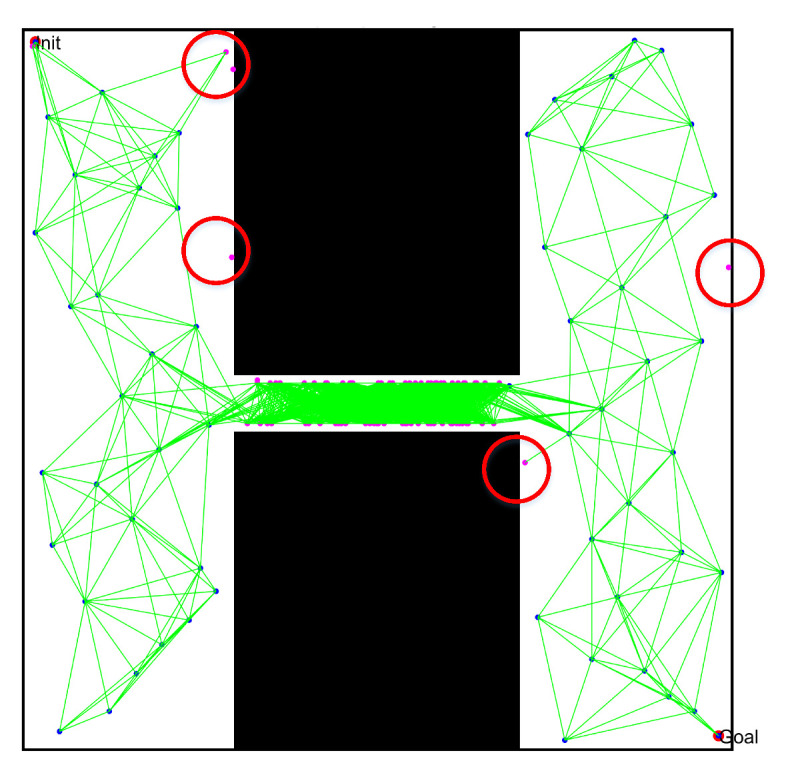
Free space construction based on the optimized PRM algorithm (Points in the red circles are not connected to other points).

After obtaining the sampling points of the free space, we used the typical connection technique in the PRM algorithm and the optimized connection technique proposed in this paper to construct line segments in free space, as shown in Figure 3b. The distribution results of the collision-free line segments are shown in Figure 16 and Figure 17. Due to the randomness of movement when optimizing the obstacle points, we directly selected the free space to observe the line segments, and found that:

(1) The optimized connecting technique can effectively avoid collision with obstacles by limiting the distance between the line segment and the nearest obstacles.
(2) Some free points may not be able to construct a collision-free line segment, due to the limitation of the maximum repulsive force, which can reduce the number of total line segments and optimize the required time for collision detection.
(3) Even if the force used for optimizing the obstacle point is random, the maximum repulsive force can effectively ensure that the free line segment is kept outside the specified range from the obstacle, especially in the narrow passage.

## 4. The Optimized Query Process

The query process of the PRM algorithm mainly obtains a successful path by querying the collision-free line segments. To reduce the required time for the query process and improve the effectiveness of the query process, we adopts the bidirectional A* algorithm, instead of the typical A* algorithm. Meanwhile, we combine a path pruning technique and a potential function to shorten the length of the final path and reduce the possibility of collision between the final path and the obstacle space. The specific principles for optimizing the query process are detailed in the following.

### 4.1. Optimizing the Query Process

After constructing the free space through the learning process, we need to solve the corresponding three performance estimated values of each free point to find the optimal path. Therefore, the complexity of solving the query process is high and the required time for solving the path planning is long. To optimize the performance of the PRM algorithm, we not only must ensure the superior performance of the original A* algorithm used in the query process, but also need to improve the speed of the local path planner. Therefore, we use the bidirectional A* algorithm to establish a local path planner both at the initial position and the final point position. The path performance evaluation functions of the two local planners can be specifically expressed as:

(11) $$\left\{\begin{array}{c} f_{Init}\left(x_{Init}\right)=g_{Init}(x_{Init})+h_{Init}\left(x_{Init}\right)\\ f_{Last}\left(x_{Last}\right)=g_{Last}(x_{Last})+h_{Last}\left(x_{Last}\right) \end{array}\right.$$

where $f_{Init}\left(\right)$ represents the evaluation value of the A* algorithm from the initial position, $f_{Last}\left(\right)$ represents the evaluation value of the A* algorithm from the final position, $g_{Init}\left(\right)$ represents the Euler distance from the free point to the initial position, $g_{Last}$ represents the Euler distance from the free node to the final position, $h_{Init}\left(\right)$ represents the Manhattan distance from the free point to the final position, and $h_{Last}\left(\right)$ represents the Manhattan distance from the node to the initial position.

**Figure 18:**
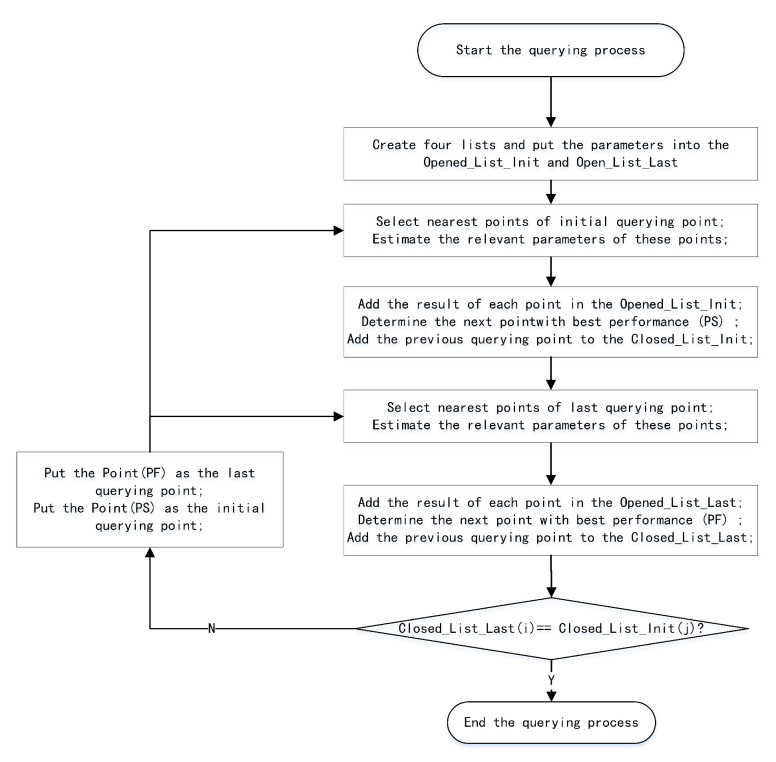
The querying process of the optimized PRM algorithm.

The typical A* algorithm generally establishes a local path planner from the initial position. Once the final position is reached, the path planning is successful. In contrast, the bidirectional A* algorithm utilizes two local path planners, from both the initial and final positions. Once a common location point exists in the two closed lists established for the bidirectional A* algorithm, the path has been successfully queried. The specific process of the bidirectional A* algorithm is shown in Figure 18. First, we created four different lists to store the different free points in the query process, including Opened_List_Last, Opened_List_Init, Closed_List_Init, and Closed_List_Last. Closed_List_Init is an empty list used to store the path nodes queried from the initial position, Closed_List_Last is an empty list used to store the path nodes queried from the final point position, Opened_List_Init assigns the initial position and corresponding cost parameters, and Opened_List_Last assigns the final position and corresponding cost parameters. Next, we query the nearest free point from the initial point and determine the next point with best performance for the initial point. In this case, we obtain the neighborhood of the initial point and determine the nearest node (PS) with the best performance. After obtaining the best point (PS), we remove the point from Opened_List_Init and add the previous point into Closed_List_Init. Then, we query the nearest free point from the goal point and determine the next point with the best performance for the goal point. In this case, we query the neighborhood of the goal point and determine the nearest node (PF) with the best performance. After obtaining the best point (PF), we remove the point from Opened_List_Last and add the previous point into Closed_List_Last. Finally, we determine whether the closed list Closed_List_Init and the closed list Closed_List_Last contain the same point. If there is a matching point, the path planning is successful; otherwise, we assign the best parameter points to the initial and final points, in order to query the next two points with the best performance.

### 4.2. Optimizing Path Pruning Technology

We can obtain a reachable path from the initial point to the target point by adopting the above optimization query method. However, there may be redundant nodes between adjacent line segments of the initial path. By observing the optimized path after removing some redundant nodes, the corrected path can still ensure the effective motion of the mobile robot. The above phenomenon occurs as any free points may select the nearest free points to connect directly, rather than using the free points outside the specified space during the connection process. Therefore, optimizing the initial path obtained by the query process is necessary.

The path pruning technology is a typical and effective method for optimizing the initial path obtained by the query process. Typical path pruning technology mainly prunes the redundant nodes of the path based on the basic principle that the sum of the two sides of a triangle must be greater than the third side. Therefore, the path pruning technique can form a new optimized path closest to the shortest path. The initial path obtained by the query process is mainly described in terms of free points. Therefore, the path pruning technique can reduce the required number of free points and optimize the memory required to describe the optimal path. In practice, we must consider whether the connection between the first node and the second node collides in the process of processing any three connected node paths. We also need to ensure that the maximum potential energy function of the optimized node connection is less than the specified value, in order to avoid possible collisions. To ensure the excellent performance of the optimal path, we optimized the pruning technology by combining the triangle principle and the potential function. The basic principle for the optimization of the three connection nodes can be specifically expressed as:

(12) $$\left\{\begin{array}{c} AC<AB+BC\\ F_{\max}\left(AC\right)\leq F_{set}\\ F\left(A\right)\leq F_{set};F\left(C\right)\leq F_{set} \end{array}\right.$$

where F() represents the repulsive force function, $F_{max}$ represents the maximum repulsive force of the line segment, A,B,C represents the three adjacent free points, and $F_{set}$ represents the specified repulsive force.

**Figure 19:**
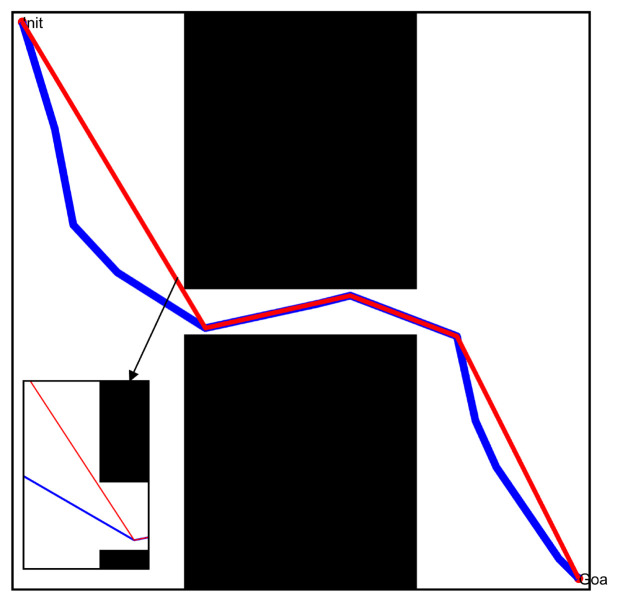
Path obtained by the typical path pruning technology.

**Figure 20:**
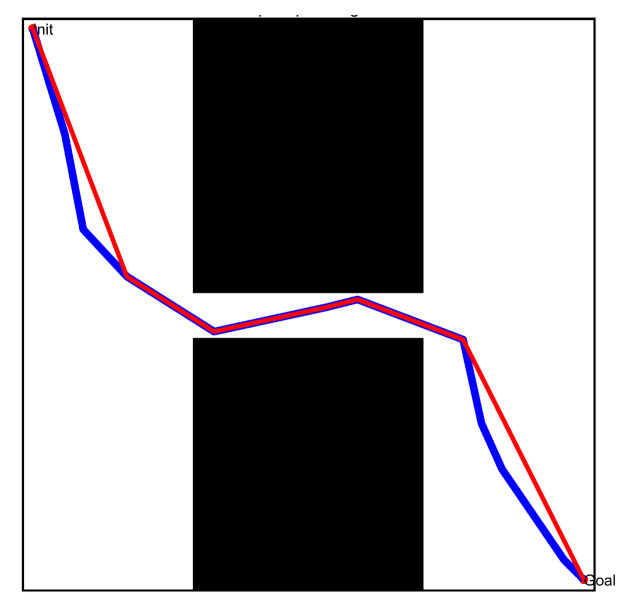
Path obtained by the optimized path pruning technology.

Assuming that the path obtained by the path query method is composed of *N* points $P_{0},P_{1},P_{2},...,P_{N}$, the path pruning technology used in this paper can be described explicitly, as in Algorithm 2. First, we initialize the data parameters, mark the initial point as Start = $P_{0}$, and set the pruning path as Path_s = $\left[P_{0}\right]$. Second, we connect the free node (Next(Path(i=2,:)) to the start point with a line segment, and perform collision detection on the line segment. When the line segment collides with an obstacle, we reset the Start point as the location of the previous point and re-construct the path. Then, we solve the maximum potential force of the overall line segment and use Formula (12) to determine whether the conditions are met. When the formula is satisfied, the free point can be optimized. In this case, we reset the Start point as the location of the previous point and re-construct the path. Otherwise, we reset the parameter *i* as i=i+1 and mark the point (Next) by the new free point. Finally, we finish path pruning when the value of *i* is equal to the total number of points in the initial path.
**Algorithm 2** Path_Opt = Optimize_Init_Path(Path, Rep_Force_Line, map).1:Start = $P_{0}$;2:Path_S = [$P_{0}$];3:**for** i = 2: length(Path) **do**4:    Next = Path(i,:);5:    **if** Collision_LineStart,Next,map== false **then**6:        **if** max(Rep_Force_Line(Next, Fins)) > $F_{set}$ **then**7:           Start = Path(i-1,:);8:           Path_S = [Path_S; Start];9:        **end if**10:    **else**11:        Start = Path(i-1,:);12:        Path_S = [Path_S; Start];13:    **end if**14:**end for**15:Path_Opt = Path_S;16:Return Path_Opt;

According to the workspace in Figure 8, we used the typical path pruning technology and the optimized path pruning technology described above to re-optimize the initial path and obtain the optimized path; the specific results are shown in Figure 19 and Figure 20. By comparing the different paths before and after optimization, it was found that: (1) The number of free points in the optimized path was significantly reduced, especially in the free space with a great area, where redundant free points were deleted, thereby effectively shortening the actual length of the path. (2) The optimized path still did not collide with the obstacles and satisfied the specified distance limit. The above results effectively illustrate that the optimized path pruning technology proposed in this paper can significantly enhance the actual performance of the initial paths obtained by the local path planner.

## 5. Simulations

In this section, we mainly verify the performance of the proposed optimized PRM path algorithm. We design various environments for path planning tasks, including single-channel path planning problems, multi-channel trajectory planning problems, and conventional environmental trajectory planning problems. The simulations designed in this section were run on a personal computer with a 3.40 GHz Intel-Core (i5-7500) CPU and 8 GB memory. The specific working map and experimental results are detailed in the following.

### 5.1. Single-Channel Path Planning Problems

**Figure 21:**
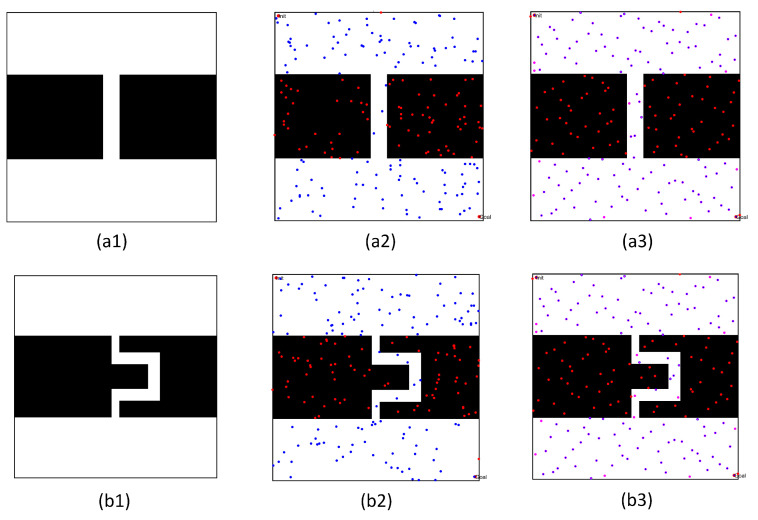
The workspaces and the sampling results of the single-channel environments: (**a1**,**b1**) Workspaces of different environments; (**a2**,**b2**) sampling points based on the random sampling method; and (**a3**,**b3**) sampling points based on the quasi-random sampling method.

In the first simulation, we chose two workspaces with single channels as simulation environments, as shown in Figure 21(a1–b1). The two workspaces have the same size (500 × 500), where the black space indicates the existence of obstacles, and the white space indicates the environment without obstacles. There is only one narrow passage in these workspaces, but that in Figure 21(a1) is a straight narrow channel, while the one in Figure 21(b1) is a winding narrow channel. To verify the effectiveness of the PRM algorithm proposed in this paper, we chose three typical PRM algorithms to conduct path planning for these workspaces. These three methods included the PRM algorithm based on random sampling, the PRM algorithm based on Gaussian sampling, and the PRM algorithm based on bridge sampling.

First, we compared the characteristics of the free points and obstacle points in these workspaces, in order to verify the superiority of the sampling principle in this paper. Therefore, these sampling points were obtained separately by the quasi-random sampling method and the random algorithm method. The specific results are shown in Figure 21(a2,b2) and (a3,b3).

By comparing the distribution results of different sampling points in Figure 21(a2,b2) and (a3,b3), the random sampling principle led to the concentration of free points in free space, such that the obstacle space could not be entirely described by the sampling points. When increasing the number of sampling points, the concentration of sampling points in free space must be more obvious, but the number of sampling points in the narrow channel may be significantly increased. However, the method proposed in this paper randomly and uniformly sampled the obstacle space and free space, thus improving the distribution characteristics of sampled points.

To demonstrate the performance of the optimized sampling algorithm, we chose the distribution of the sampling points in the narrow channel and path planning results in two workspaces for analysis and comparison with the other PRM algorithms considered in this paper. First, we selected the different PRM algorithms to solve the path planning problem, and each method was performed 20 times. Then, we used statistical methods to obtain various parameters for the four PRM algorithms in the different workspaces, including the mean number of all sampling points (Ns), the mean number of free points (Nf), the mean number of the free point in the narrow channel (Nc), the mean time required for completing the sampling point (Ts), the mean proportion of free points in the narrow passage to the total free points (Pf), the mean time required for constructing free space (TL), and the success rate of path planning (Suc). These results are provided in Table 1. Finally, we recorded the query process time, as given in Table 2, where TQ1 indicates the time taken to obtain the path by a typical query method, and TQ2 indicates the time taken to obtain the path using the optimized query method.

(1) By setting the same number of sampling points, the optimized sampling technology proposed in this paper can increase the number of free spaces to find the collision-free path. The PRM algorithm based on random sampling principle directly sets the total number of free points and the obstacle points. The other two PRM algorithms mainly directly set the number of free points.
(2) By increasing the number of sampling points, the number of sampling points in the narrow channel can be rapidly increased. This is because the sampling optimization technique in this paper can turn obstacle points into free points in narrow passages.
(3) By comparing the number of free points in the overall workspace to that in the narrow channel, the quasi-random sampling principle and obstacle point optimization technique proposed in this paper can significantly increase the number of sampling points in the narrow channel.
(4) By comparing the success rate of finding the path, it was found that the method proposed in this paper can enhance the success rate of path planning, even when using a lower number of sampling points than the other PRM algorithms.
(5) By comparing the required time for the query process shown in Table 2, it can be seen that the method proposed in this paper took less time to find an effective path, effectively increasing the efficiency of the PRM algorithm.

**Figure 22:**
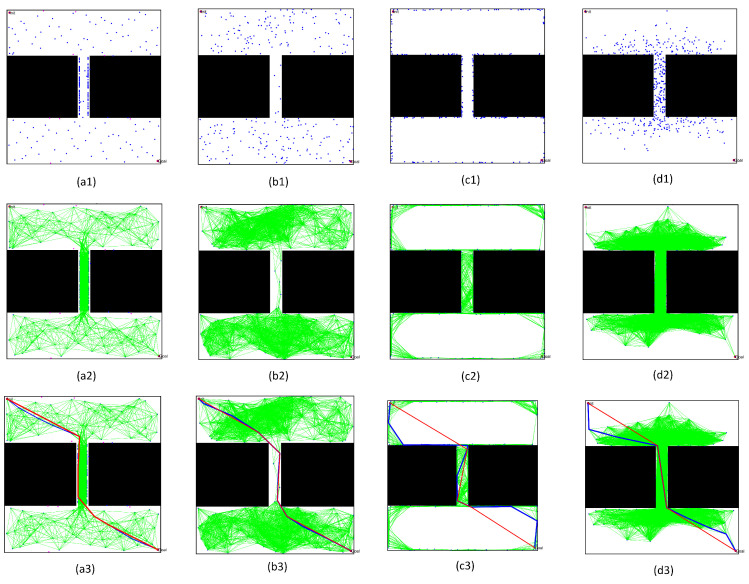
Results of various PRM algorithms in the environment with a straight channel: (**a1**–**a3**) based on the sampling method proposed in this paper; (**b1**–**b3**) based on random sampling method; (**c1**–**c3**) based on Gaussian sampling method; and (**d1**–**d3**) based on Bridge sampling method.

**Figure 23:**
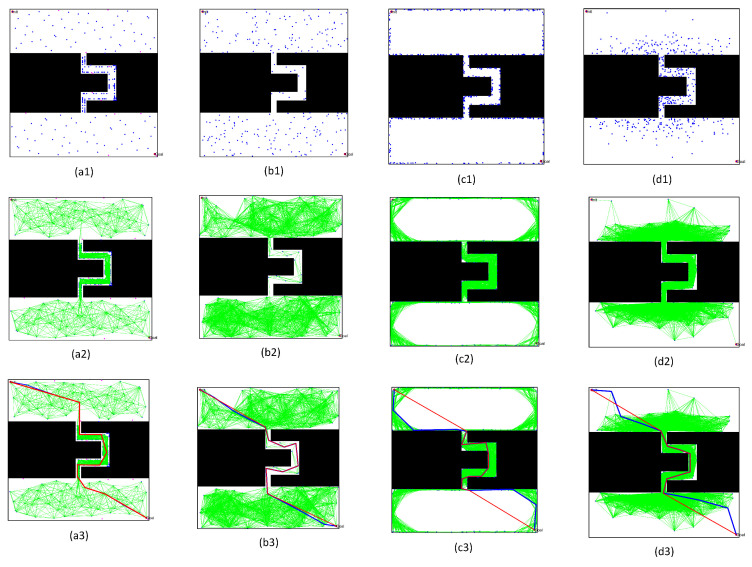
Results of various PRM algorithms in the environment with a non-straight channel: (**a1**–**a3**) based on the sampling method proposed in this paper; (**b1**–**b3**) based on random sampling method; (**c1**–**c3**) based on Gaussian sampling method; and (**d1**–**d3**) based on Bridge sampling method.

We chose the above four typical PRM algorithms to intuitively describe the superior performance of the algorithm proposed in this paper. The typical PRM algorithms included the PRM based on random sampling, the PRM based on Gaussian sampling, and the PRM based on bridge sampling. To compare the performance of these PRM algorithms, we set the number of sampling points as 200, and obtained the actual distribution of the sampling point and the path planning results. By comparing the sampling results, it was found that:

(1) Comparing the free space construction results in Figure 22(a1–d1) and Figure 23(a1–d1), the optimized sampling technology in this paper directly increased the sampling number in the narrow passages. Meanwhile, the collision-free local path in free space ensured that the shortest distance to obstacles was within the specified range.
(2) By comparing the path query results in Figure 22(a3–d3) and Figure 23(a3–d3), the optimized query algorithm proposed in this paper could effectively obtain the initial path, showing no collision with the obstacle space. The distance from the nearest obstacle satisfied the specified distance constraint, ensuring the basic performance of the optimized path.
(3) By comparing the results before and after optimizing the initial path in Figure 22(a3–d3) and Figure 23(a3–d3), the path optimization technology used in this paper could effectively delete redundant points in the initial path, meet the distance limit for the obstacles, shorten the length of the path, and optimize the actual.

### 5.2. Multi-Channel Path Planning Problems

**Figure 24:**
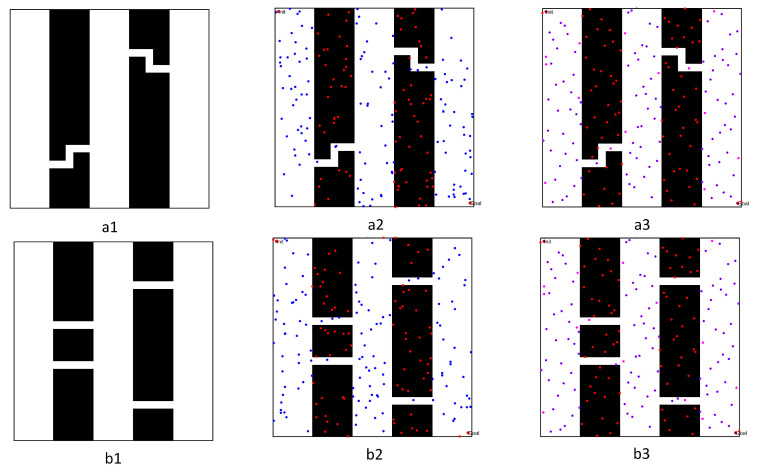
The workspace and sampling results for environments with multiple narrow channels: (**a1**,**b1**) The workspaces of different environments; (**a2**,**b2**) sampling points based on the random sampling method; and (**a3**,**b3**) sampling points based on the quasi-random sampling method.

After completing the single-channel path planning problem, we designed a more complex path planning problem involving two multi-channel environments, in order to demonstrate the effectiveness of the proposed PRM algorithm. In these experiments, the size of the environments was still 500 × 500, where the first map was composed of two winding channels, and the second workspace included three narrow channels, as shown in Figure 24. To prove the effectiveness of the optimized PRM algorithm proposed in this paper, we chose one typical PRM algorithm and two optimized PRM algorithms to solve the path planning problem for a mobile robot in complex environments with multiple narrow channels.

We set the number of samples to be 100, 200, or 300, and used the three PRM algorithms to perform sampling in different workspace. Different results related to the learning process and query process are summarized below, and can be seen in Table 3 and Table 4; the meanings of the variable in the two tables are the same as in the first experiment.

According to the comparison between the number of sampling points in the narrow channel and the actual results of different path planning problems in Table 3 and Table 4, it was found that:

(1) By increasing the number of sampling points, the number of free points in the narrow channel increased significantly in these PRM algorithms. However, the free points in the narrow channel could be increased directly by optimizing the obstacle points after obtaining the overall sampling points. Therefore, the optimized PRM algorithm proposed in this paper increased the number more than the other algorithms.
(2) As the number of sampling points increased, the required time for the learning and query processes increased in these PRM algorithms. Nevertheless, the optimized PRM algorithm proposed in this paper could successfully complete path planning with a lower number of sampling points.
(3) By comparing the success rate with similar time for constructing the free space, the solution success rate of the proposed algorithm was higher than that of the other algorithms within a similar time. For example, comparing Task4 with 100 points based on the new algorithm and 300 points based on the other algorithms in Table 3, the success rate of the optimized PRM algorithm reached 100%, while the success rate of the other algorithms was less than 80%.
(4) By comparing the required time for the learning process, the sampling time was higher than for the other PRM algorithms. However, the other PRM algorithms could not effectively solve the path planning problem in a complex environment. For example, by setting the same initial number sampling points as 300 in the various PRM algorithms for solving Task3 and Task4, the corresponding solution success rate of the optimized PRM algorithm reached 100%, while the other PRM algorithms potentially could not solve the problem.
(5) By comparing the required time for the query processes, as shown in Table 4, the required time for the optimized query process was shorter than that of other PRM methods. The optimized query process proposed in this paper can effectively improve the required time for finding the initial path in the narrow channel.

**Figure 25:**
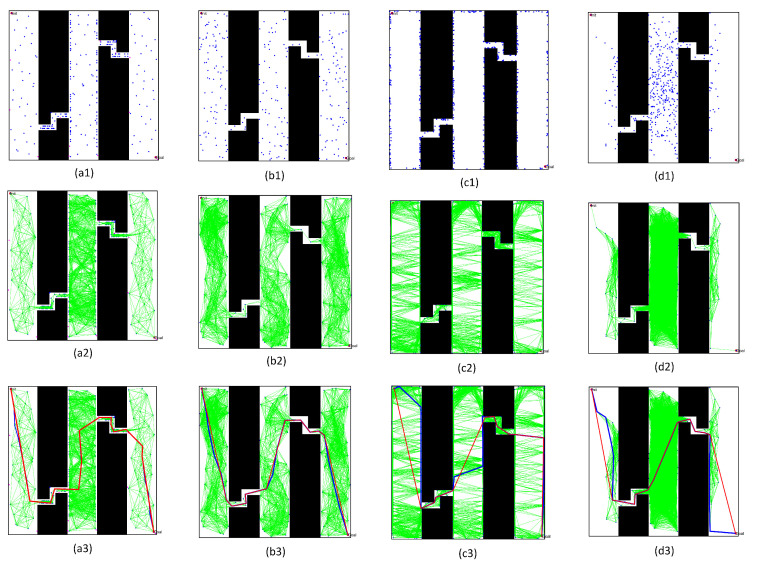
Results of various PRM algorithms in the environment with two narrow channels: (**a1**–**a3**) Based on the sampling method proposed in this paper; (**b1**–**b3**) based on random sampling method; (**c1**–**c3**) based on Gaussian sampling method; and (**d1**–**d3**) based on Bridge sampling method.

**Figure 26:**
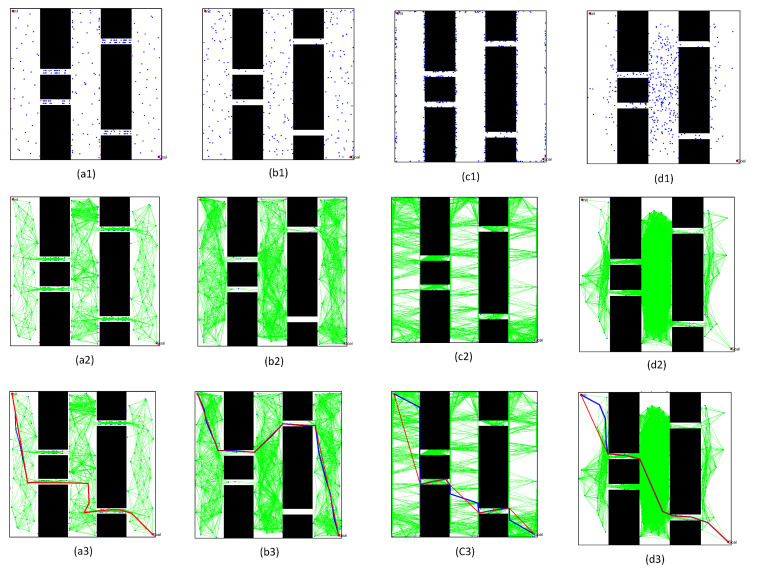
Results of various PRM algorithms in the environment with four narrow channels: (**a1**–**a3**) Based on the sampling method proposed in this paper; (**b1**–**b3**) based on random sampling method; (**c1**–**c3**) based on Gaussian sampling method; and (**d1**–**d3**) based on Bridge sampling method.

To directly observe the results of the three PRM algorithms, we set the number of sampling points to 300 and performed path planning in the complex workspaces with multi-narrow channels, as shown in Figure 25 and Figure 26. Comparing and analyzing the specific results, we found that:

(1) The density of free points obtained by Gaussian sampling was still near the obstacles, but the PRM algorithm based on bridge sampling reduced the density of free points in the narrow channels, due to the existence of wide channels in the environment. Similarly, the optimized PRM algorithm in this paper reduced the number of free points in the narrow channels due to the existence of wide channels. Compared with other methods, the number of free points obtained by the optimized PRM algorithm in the narrow channels was still the largest, as shown in Figure 25(a1) and Figure 26(a1).
(2) By comparing the distribution of the free points and collision-free line segments in Figure 25(a2–d2) and Figure 26(a2–d2), the optimized PRM algorithm proposed in this paper effectively avoided collision with obstacles, thus improving the performance of the local path.
(3) By comparing the paths obtained by the query process in Figure 25(a3–d3) and Figure 26(a3–d3), there was a great difference between the final path obtained by the optimized PRM algorithm and the final paths obtained by other PRM algorithms, due to the deletion of different redundant points. However, the optimized PRM algorithm effectively ensured the a certain distance from the nearest obstacle boundary was maintained. Therefore, the path optimization technology proposed in this paper can effectively improve the performance of the initial path obtained in the query process.

### 5.3. Path Planning of Normal Workspace

**Figure 27:**
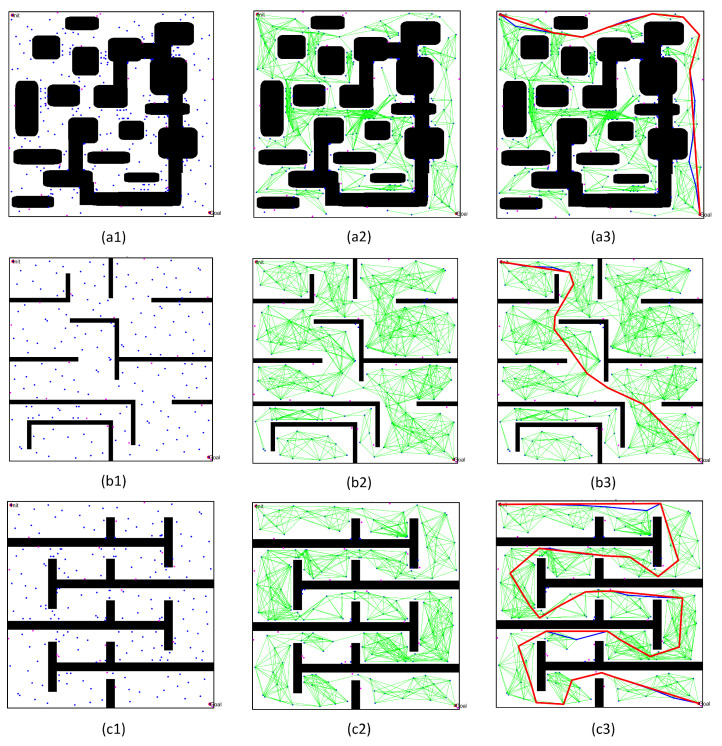
The results of the optimized PRM algorithm in three normal workspaces: (**a1**–**a3**) Sampling points in the three environments; (**b1**–**b3**) free space construction results in the three environments; and (**c1**–**c3**) query process results in the three environments.

After completing path planning in the single- and multi-channel scenarios, we verified the effectiveness of the optimized PRM algorithm in normal environments. Therefore, we selected three conventional workspaces to solve the path planning problem in a specific environment, as shown in Figure 27. These three workspaces were composed of multiple obstacles. The first map included multiple significant obstacles, with a working environment more complex than the other workspaces; meanwhile, the second and third maps included multiple grid obstacles. The size of the three workspaces was 500 × 500, the initial position was (10,10), and the final point position was (490,490). We use the optimized PRM algorithm proposed in this paper to solve the path planning problem, effectively demonstrating the effectiveness of the PRM algorithm proposed in this paper in normal environments.

Figure 27 represents the critical results for solving the path planning of the three regular workspaces. In these figures, the blue dots represent free points, while the pink dots are adjacent points. Otherwise, the green line segment is the collision-free line segment in free space, the blue path is the initial path successfully obtained by the query process, and the red path represents the final optimized path. By analyzing the results of path planning for the three workspaces, we found that:

(1) Comparing the distribution and the density of sampling points in Figure 27(b1,c1), the number of obstacles increased significantly, but the volume of obstacles decreased. Fewer free points could be obtained in the narrow channel by obstacle point optimization technology, leading to significantly reduced sampling probability in the workspace. However, the quasi-random sampling method can guarantee enough sampling points to construct the free space of the normal workspace effectively.
(2) Comparing the actual distribution of the sampling points in Figure 27(a1), the external shape of the obstacle had changed. However, the obstacle point optimization technology could still transform the obstacle points into free points, effectively increasing the number of free points near the boundary of narrow channels. It also effectively increased the number of sampling points in free space. Therefore, the PRM algorithm proposed in this paper can still effectively deal with the path planning problem in normal environments.
(3) Comparing the initial path and the optimized path in Figure 27(a3,c3), the optimized query process proposed in this paper obtained an initial path far from the obstacle space. Meanwhile, the optimized path pruning technology effectively removed the redundant points of the initial path without changing the distance to the obstacle, thus shortening the length of the path.

## 6. Conclusions

In this paper, we proposed a novel PRM which can effectively solve the problem of path planning for mobile robots in a complex environments with narrow channels. First, we expanded the obstacle using the diagonal distance of the robot to ensure the existence of all narrow channels in the working map. In addition,, we obtained the sampling point based on the quasi-random sampling principle and reclassified the sampling point type, which can optimize the distribution of random points and ensures the safety of the free points. Second, the obstacle points were transformed from the obstacle space to the narrow passage through use of the potential energy function and the characteristics of the narrow channel, thus improving the density of the sampling points in the narrow passage. Meanwhile, the connection line in the free space was optimized to improve the local motion path obtained by the PRM algorithm in the narrow passage. Third, the bidirectional A* algorithm was chosen, instead of the typical local path planner, in order to reduce the time required by the query process. Furthermore, the potential energy function and path pruning were effectively combined to optimize the initial path obtained by the query process. Finally, we conducted many experiments, in order to demonstrate that the PRM algorithm proposed in this paper can reduce the collision probability of the robot while optimizing the length of the overall path, thus effectively solving the path planning problem for workspaces with narrow environments.

The new PRM algorithm proposed in this paper mainly realizes the path planning problem for mobile robots in global static state. However, the surrounding information of the complex environment typically changes with time in actual workspaces. Therefore, solving the path planning problem for dynamic environments by effectively combining the PRM algorithm and the motion of obstacles is considered a valuable future research direction.

## Figures and Tables

**Table 1:** Various parameters of four PRM algorithms in the single-channel environments.

Map	Ns ^1^	Method ^2^	Nf ^3^	Nc ^4^	Ts ^5^	Pf ^6^	TL ^7^	Suc ^8^
1	100	Random	62.9000	2.7500	0.0829	4.37%	0.0490	25%
		Gaussian	102	18.050	0.0934	17.6%	0.0661	5%
		Bridge	102	33.400	0.0854	32.7%	0.3039	0%
		Paper	136.8	70.700	0.2825	51.6%	0.6258	100%
	200	Random	123.8	6.3500	0.1324	5.13%	0.1569	50%
		Gaussian	202	34.7500	0.1655	17.2%	0.2192	55%
		Bridge	202	67.2000	0.1341	33.2%	0.3223	35%
		Paper	270.7	139.650	0.5442	51.5%	2.4632	100%
	300	Random	186.2	9.1000	0.1955	4.8%	0.3189	95%
		Gaussian	302	52.6500	0.2078	17.4%	0.4571	80%
		Bridge	302	99.1000	0.1975	32.8%	0.3321	45%
		Paper	408.2	214.900	0.8185	52.6%	2.7158	100%
2	100	Random	65.0500	3.5500	0.0684	5.45%	0.0516	0%
		Gaussian	102	27.2500	0.0799	26.7%	0.0609	0%
		Bridge	102	32.0500	0.0694	31.4%	0.3407	15%
		Paper	136.7	70.5500	0.2835	51.6%	0.2629	100%
	200	Random	128.4	7.4500	0.1324	5.79%	0.1585	0%
		Gaussian	202	54.9000	0.1442	27.2%	0.2002	25%
		Bridge	202	67.2500	0.1328	33.2%	0.3337	30%
		Paper	272.3	140.400	0.5840	51.5%	0.9884	100%
	300	Random	186.6	12.7000	0.2002	6.81%	0.3197	30%
		Gaussian	302	84.6500	0.2073	28.02%	0.4124	55%
		Bridge	302	100.550	0.1961	33.2%	0.3379	35%
		Paper	412.1	216.650	0.8806	52.5%	2.3390	100%

^1^ the mean number of all sampling points. ^2^ the PRM algorithm with different sampling principles. ^3^ the mean number of free points. ^4^ the mean number of the free point in the narrow channel. ^5^ the mean time required for completing the sampling point. ^6^ the mean proportion of free points in the narrow passage to the total free points. ^7^ the mean time required for constructing free space. ^8^ the success rate of path planning in 20 experiments.

**Table 2:** Parameters of different query process in the single-channel environments.

Map	Ns ^1^	TQ1 ^2^			TQ2 ^3^			Map	Ns ^1^	TQ1 ^2^			TQ2 ^3^		
		Max	Min	Mean	Max	Min	Mean			Max	Min	Mean	Max	Min	Mean
1	100	0.1435	0.1114	0.1257	0.0365	0.0241	0.0285	2	100	0.0766	0.0495	0.0582	0.0198	0.0036	0.0103
	200	0.3417	0.2621	0.2927	0.1131	0.0995	0.1056		200	0.2059	0.1593	0.1818	0.0426	0.0339	0.0371
	300	0.6049	0.4956	0.5576	0.3008	0.2530	0.2735		300	0.4000	0.3017	0.3380	0.0928	0.0788	0.0860

^1^ the mean number of all sampling points. ^2^ the time required for the query process in the traditional PRM algorithm. ^3^ the time required for the query process in the optimized PRM algorithm.

**Table 3:** Various parameters of four PRM algorithms in the multi-channel environments.

Map	Ns ^1^	Method ^2^	Nf ^3^	Nc ^4^	Ts ^5^	Pf ^6^	TL ^7^	Suc ^8^
3	100	Random	61.4000	0.8500	0.0730	1.38%	0.0436	0%
		Gaussian	102	14.350	0.0868	14.1%	0.0589	0%
		Bridge	102	5.1000	0.0736	5.00%	0.0392	0%
		Paper	139	39.350	0.2680	28.3%	0.2148	25%
	200	Random	121.45	2.7000	0.1358	2.22%	0.1292	0%
		Gaussian	202	28.100	0.1460	13.9%	0.1847	5%
		Bridge	202	9.5500	0.1355	4.73%	0.0406	5%
		Paper	276	73.900	0.5218	26.7%	0.8879	90%
	300	Random	183.8	5.8000	0.2085	3.15%	0.2625	0%
		Gaussian	302	48.950	0.2072	16.2%	0.3872	30%
		Bridge	302	13	0.1999	4.30%	0.0475	15%
		Paper	413	103.050	0.8010	25.0%	1.9799	100%
4	1	Random	61	2.2000	0.0748	3.61%	0.0485	10%
		Gaussian	102	21.200	0.0881	20.7%	0.0585	0%
		Bridge	102	7.1000	0.0746	6.96%	0.0588	0%
		Paper	135	62.850	0.2333	46.5%	0.2289	100%
	200	Random	125	5	0.1377	4.00%	0.1324	40%
		Gaussian	202	41.3500	0.1557	20.5%	0.1806	10%
		Bridge	202	14.3000	0.1372	7.08%	0.0691	25%
		Paper	274	122.200	0.4615	44.6%	0.8141	100%
	300	Random	185	8.6000	0.2010	4.64%	0.2777	75%
		Gaussian	302	64.5500	0.2302	21.4%	0.3694	45%
		Bridge	302	24	0.2027	7.95%	0.0791	65%
		Paper	413	192	0.6893	46.5%	1.8628	100%

^1^ the mean number of all sampling points. ^2^ the PRM algorithm with different sampling principles. ^3^ the mean number of free points. ^4^ the mean number of the free point in the narrow channel. ^5^ the mean time required for completing the sampling point. ^6^ the mean proportion of free points in the narrow passage to the total free points. ^7^ the mean time required for constructing free space. ^8^ the success rate of path planning in 20 experiments.

**Table 4:** Parameters of different query process in the multi-channel environments.

Map	Ns ^1^	TQ1 ^2^			TQ2 ^3^			Map	Ns ^1^	TQ1 ^2^			TQ2 ^3^		
		Max	Min	Mean	Max	Min	Mean			Max	Min	Mean	Max	Min	Mean
3	100	0.0647	0.0074	0.0330	0.02284	0.0008	0.0050	4	100	0.0530	0.0410	0.0472	0.0208	0.0081	0.0098
	200	0.2708	0.0261	0.2179	0.03967	0.0048	0.0286		200	0.2110	0.1280	0.1900	0.0413	0.0331	0.0358
	300	0.5700	0.4416	0.4933	0.07858	0.0106	0.0714		300	0.2941	0.2548	0.2783	0.0735	0.0648	0.0690

^1^ the number of sampling points. ^2^ the time required for the query process in the traditional PRM algorithm. ^3^ the time required for the query process in the optimized PRM algorithm.

## Data Availability

Not applicable.
